# Dissecting the regulation of pollen tube growth by modeling the interplay of hydrodynamics, cell wall and ion dynamics

**DOI:** 10.3389/fpls.2014.00392

**Published:** 2014-08-11

**Authors:** Junli Liu, Patrick J. Hussey

**Affiliations:** School of Biological and Biomedical Sciences, Durham UniversityDurham, UK

**Keywords:** pollen tube growth, mathematical modeling, oscillatory dynamics, interplay of hydrodynamics, cell wall and ion dynamics, regulation coefficients

## Abstract

Hydrodynamics, cell wall and ion dynamics are all important properties that regulate pollen tube growth. Currently, the two main pollen tube growth models, the cell wall model and the hydrodynamic model do not appear to be reconcilable. Here we develop an integrative model for pollen tube growth and show that our model reproduces key experimental observations: (1) that the hypertonic condition leads to a much longer oscillatory period and that the hypotonic condition halves the oscillatory period; (2) that oscillations in turgor are experimentally undetectable; (3) that increasing the extracellular calcium concentration or decreasing the *p*H decreases the growth oscillatory amplitude; (4) that knockout of Raba4d, a member of the Rab family of small GTPase proteins, decreases pollen tube length after germination for 24 h. Using the model generated here, we reveal that (1) when cell wall extensibility is large, pollen tube may sustain growth at different volume changes and maintain relatively stable turgor; (2) turgor increases if cell wall extensibility decreases; (3) increasing turgor due to decrease in osmolarity in the media, although very small, increases volume change. However, increasing turgor due to decrease in cell wall extensibility decreases volume change. In this way regulation of pollen tube growth by turgor is context dependent. By changing the osmolarity in the media, the main regulatory points are extracellular osmolarity for water flow and turgor for the volume encompassed by the cell wall. However, if the viscosity of cell wall changes, the main regulatory points are turgor for water flow and wall extensibility for the volume encompassed by the cell wall. The novel methodology developed here reveals the underlying context-dependent regulatory principle of pollen tube growth.

## Introduction

The pollen tube is a model system for the study of tip growth in plants (Feijo et al., 2001). Essential features of pollen tube growth are polarization of ion fluxes, intracellular ion gradients, and oscillating dynamics. The directional, polar tip growth of a pollen tube involves a highly coordinated movement of vesicles bearing large amounts of new cell wall and plasma membrane materials to be integrated into the growing apical region. These features of pollen tube growth are regulated by a wide range of spatiotemporally organized processes such as exocytosis and endocytosis, actin cytoskeleton reorganization, cell wall deposition, intracellular signaling, and ion fluxes. For example, experimental manipulation of tip associated calcium ion gradients can result in re-polarization (Malho et al., 1994) suggesting an intimate relationship between ion flux and pollen tube growth.

When a pollen tube grows, new cell wall and plasma membrane materials have to be integrated into the growing apical region and water has to flow into the pollen tube. Since the volume encompassed by the cell wall (cell wall chamber) and the volume of cellular solution in a pollen tube are the same, the relative change in the volume of water and the relative change in cell wall chamber are equal during pollen tube growth (Ortega, 1994, 2010). Therefore, both cell wall properties and hydrodynamics are closely associated with pollen tube growth. Any molecular component affecting cell wall properties and/or hydrodynamics must have a role in pollen tube growth. Currently, there are two main models of pollen tube growth. The cell wall model considers that cell wall mechanical properties control growth (Winship et al., 2010, 2011) and the hydrodynamic model suggests that the intracellular pressure, turgor, controls growth (Zonia and Munnik, 2011). For the cell wall model, it is suggested that the cell wall sets the pace for pollen tube growth; the main experimental evidence being that the stiffness of the cell wall is inversely correlated with growth rate, and that there are no rapid and large-scale turgor changes during growth (Winship et al., 2010, 2011). For the hydrodynamic model, hypertonic and hypotonic osmolarity was shown to cause the pollen tube apical area to shrink and swell respectively, and these changes correspond to the doubling and halving of growth rate oscillatory periods respectively compared to the oscillatory period of the isotonic growth condition (Zonia et al., 2006; Zonia and Munnik, 2007; Winship et al., 2011). Therefore, it was suggested (Zonia and Munnik, 2004) that growth rate oscillations are regulated by hydrodynamics.

There are no current mathematical models that can reconcile the cell wall and hydrodynamic models for pollen tube growth (Zonia and Munnik, 2004, 2007, 2011; Zonia et al., 2006; Winship et al., 2010, 2011; Kroeger and Geitmann, 2012). Key aspects of the most recent models have been reviewed by Kroeger and Geitmann (2012). In particular, Hill et al. (2012) developed an osmotic model for pollen tube growth. Their model predicts that osmotic permeability is restricted to a constant area near the tip, which was experimentally confirmed. Importantly, their model shows that the turgor pressure has two opposing effects—“controlling the water entry; and controlling the area expansion of the tip wall polymers (pectin) which translates into new cell volume” (Hill et al., 2012). However, this model assumes osmotic pressure is an independent parameter, and it could not generate oscillatory dynamics, which is one of the main features of pollen tube growth. Based on a model for calcium dependent oscillatory growth in pollen tubes (Kroeger et al., 2008), Kroeger et al. (2011) developed a model to investigate the relationship between growth rate and turgor. Their model shows that changes in the global turgor do not influence the average growth rate in a linear manner. However, this model does not consider water permeability and therefore it could not investigate the effects of osmolarity on pollen tube growth. Liu et al. (2010) developed a model to investigate the dynamics of four major ions (Ca^2+^, K^+^, Cl^−^, H^+^) in pollen tube growth. This model shows that tip and shank of a pollen tube forms an integrative system generating oscillations at the tip. However, this model does not include water permeability and cell wall deposition. Moreover, a model, which considers vesicle trafficking only and does not show any oscillations, shows that vesicle trafficking can be directly correlated with the pollen tube growth rate (Kato et al., 2010). In addition, other models for pollen tube growth include a detailed model of cell wall mechanics that proposes a negative feedback between growth rate and vesicle secretion (Rojas et al., 2011); a model that investigates the role of cell wall ageing (Eggen et al., 2011); and a model that investigates the role of calcium in participating in feedback regulation of the oscillating ROP1 Rho GTPase (Yan et al., 2009). Therefore, although different models for pollen tube growth exist, they have not integrated hydrodynamics, cell wall and ion dynamics into a whole system. In particular, “independent parameters” are arbitrarily chosen (i.e., assuming that a parameter such as cellular osmotic pressure can be arbitrarily changed by the modelers) to analyse pollen tube growth. In reality, during pollen tube growth, the relative change in the volume of water and cell wall chamber is always equal. Moreover, the pollen tube itself regulates those “independent parameters,” so that their changes must follow fundamental laws governing pollen tube growth. For example, the pollen tube itself regulates cellular osmotic pressure by regulating ion and other concentrations. Due to the lack of a methodology to study the regulation of pollen tube growth, how pollen tube growth is regulated is unknown (Liu and Hussey, 2011; Kroeger and Geitmann, 2012). In this work, based on a variety of experimental information, we use a systems model to develop insights into pollen tube growth.

## Results

### A pollen tube tip growth model with intrinsic coupling of hydrodynamics, cell wall and ion dynamics

Osmolarity in the media may change by changing the concentrations of ions or other components e.g., mannitol. Experimentally, it has been shown that ion dynamics and growth dynamics change when the osmolarity changes in the media (Messerli and Robinson, 2003; Zonia and Munnik, 2004, 2007, 2011; Zonia et al., 2006; Kroeger et al., 2011). Importantly, to understand the relationship between osmolarity in the media and pollen tube growth dynamics, integration of all the biological processes involved is required as the pollen tube regulates its growth as a whole (Liu and Hussey, 2011; Kroeger and Geitmann, 2012).

**Figure 1:**
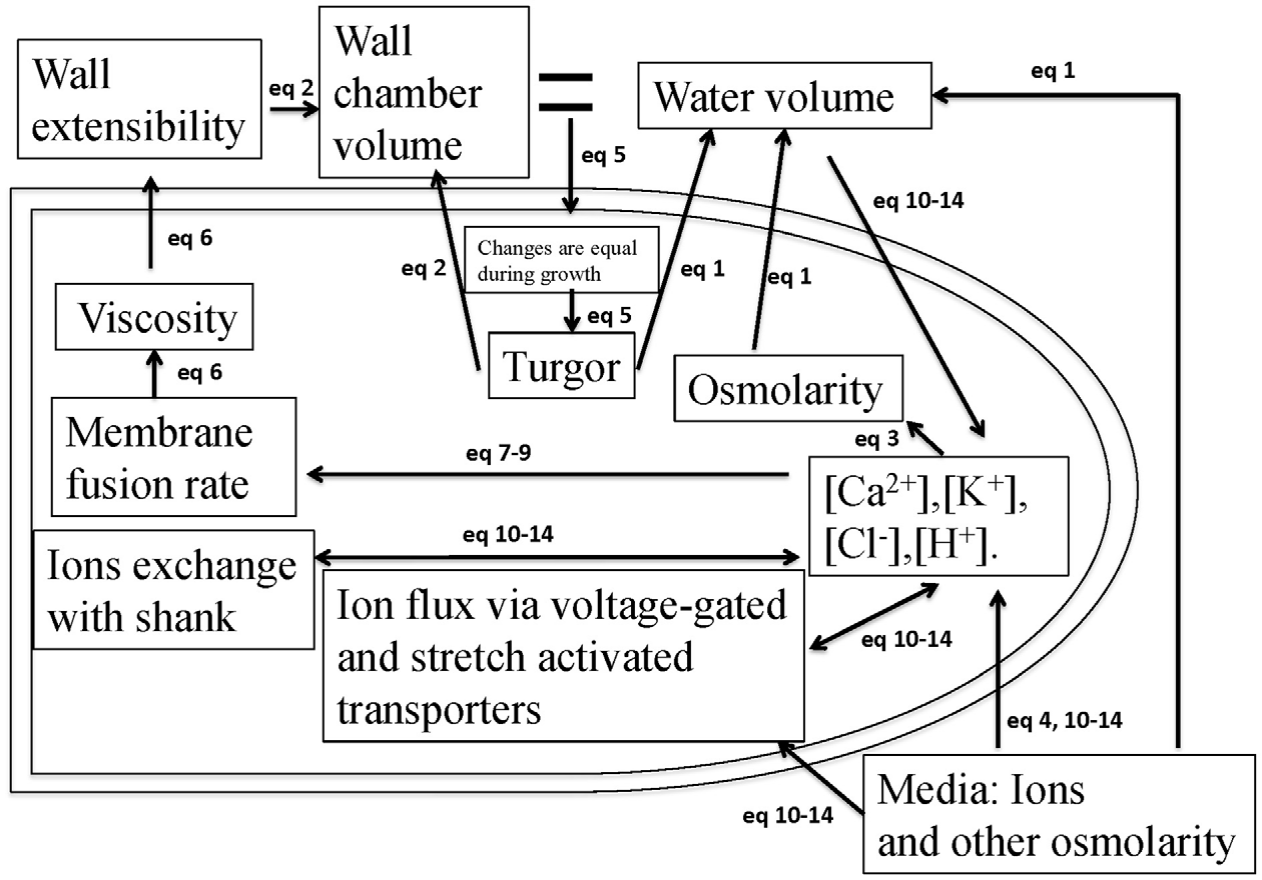
**A schematic description of the model that couples hydrodynamics, cell wall and ion dynamics**. During pollen tube growth, the relative change in the volume of water and the volume encompassed by the cell wall (cell wall chamber) is equal (Ortega, 2010), and turgor is generated. Calcium affects vesicle secretion rate that affects cell wall viscosity, which further affects cell wall extensibility. Both cell wall extensibility and turgor affect the volume change of the cell wall chamber (Kroeger et al., 2011; Hill et al., 2012). Both turgor and osmotic pressure affect the volume of water (Winship et al., 2010; Hill et al., 2012). Ion concentrations affect cellular osmotic pressure. The equations used to describe these processes are included in the Materials and Methods Section. The parameters and their links with experimental data are included in SI-Appendix.

During pollen tube growth, the relative change in the volume of water and cell wall chamber is equal (Ortega, 1994, 2010). The model in Figure 1 describes how the pollen tube itself regulates its growth by integrating hydrodynamics, cell wall and ion dynamics. The equations used to describe these processes are included in the Materials and Methods Section. The parameters and their links with experimental data are included in SI-Appendix.

### Modeling results are in agreement with experimental observations

Experimental data show that pollen tube growth is sensitive to changes in osmolarity in the media. Hypo-osmotic treatment causes cell radius swelling, while hyperosmotic treatment causes cell radius shrinking. Importantly, the period of the oscillations in growth rate is dependent on osmolarity in the media. Compared to the control (isotonic condition) with an oscillatory period of ca. 50 s, hyperosmotic treatment increases the period to ca. 100 s, and hypo-osmotic treatment decreases the period to ca. 25 s (Zonia and Munnik, 2004, 2007, 2011; Zonia et al., 2006).

**Figure 2:**
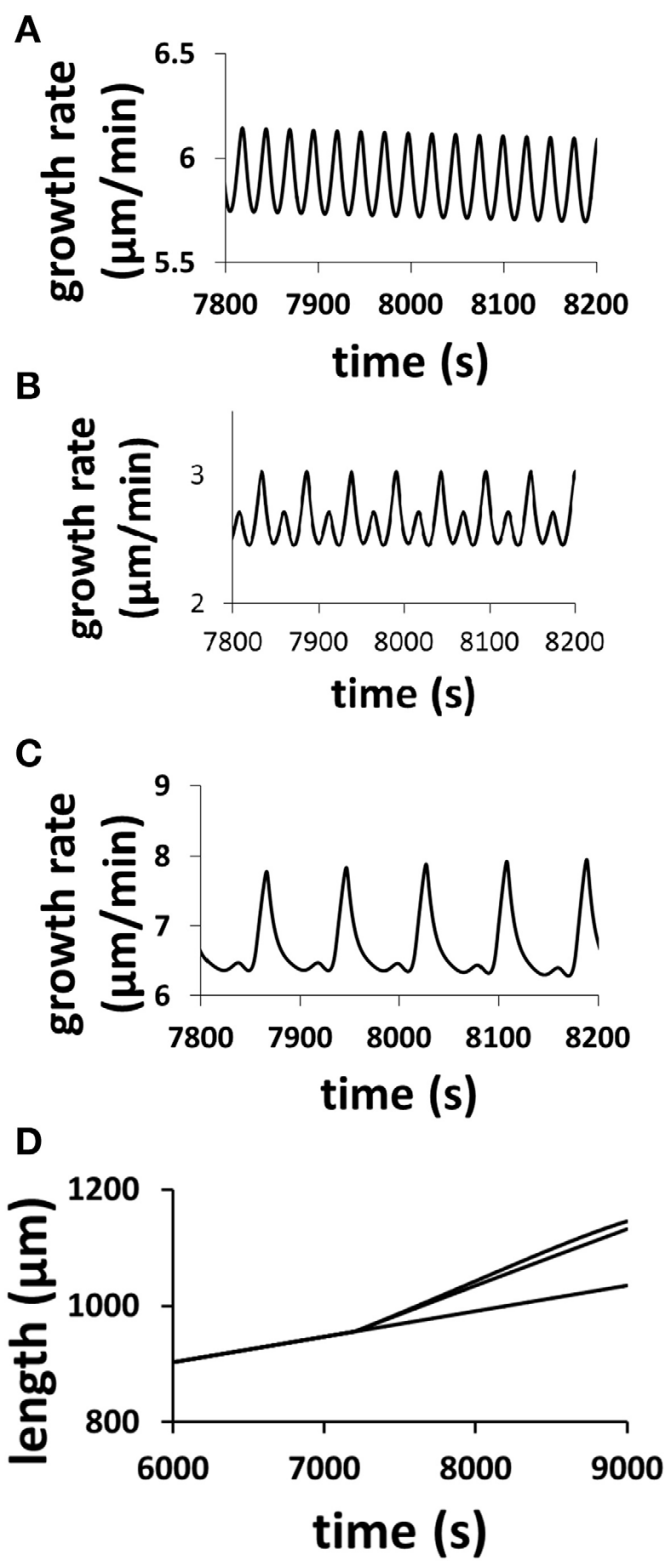
**Modeling results reproduce the dependence of oscillatory periods of growth rate and pollen tube length on osmolarity in the media**. Pollen tube grows in a media of 0.36 Osm. At time = 7200 s, osmolarity in the media changes to **(A)** 0.18 Osm; **(B)** 0.36 Osm (i.e., remaining unchanged); **(C)**: 1.16 Osm following experiments (Zonia and Munnik, 2004, 2007, 2011; Zonia et al., 2006). **(D)**: Panel **(D)** compares pollen tube length for the above three media conditions (top: 1.16 Osm; middle: 0.18 Osm; bottom: 0.36 Osm). In **(A)**, the pollen tube radius linearly increases from 5 to 5.5 μm from 7200 to 9000 s. In **(B)**, the pollen tube radius does not change (5 μm). In **(C)**, the pollen tube radius linearly decreases from 5 to 3.5 μm from 7200 to 9000 s. In experiments, Zonia and Munnik (2004) show that, when osmolarity in the media decreases from 0.36 to 0.18 Osm, pollen tube radius increases. When osmolarity in the media increases from 0.36 to 1.16 Osm, pollen tube radius decreases.

The model (Figure 1), which intrinsically couples hydrodynamics, cell wall and ion dynamics, reproduces the dependence of oscillatory dynamics on the osmolarity in the media. Figure 2 shows the dependence of growth rate and pollen tube length on osmolarity in the media. In isotonic media (0.36 Osm), oscillations emerge with a period of ca. 50 s. When the media becomes hypotonic (0.18 Osm), the pollen tube grows with a much shorter oscillatory period (ca. 25 s). However, when the media is hypertonic (1.16 Osm), oscillatory periods are much longer (ca. 87 s) (Zonia and Munnik, 2004, 2007, 2011; Zonia et al., 2006).

**Figure 3:**
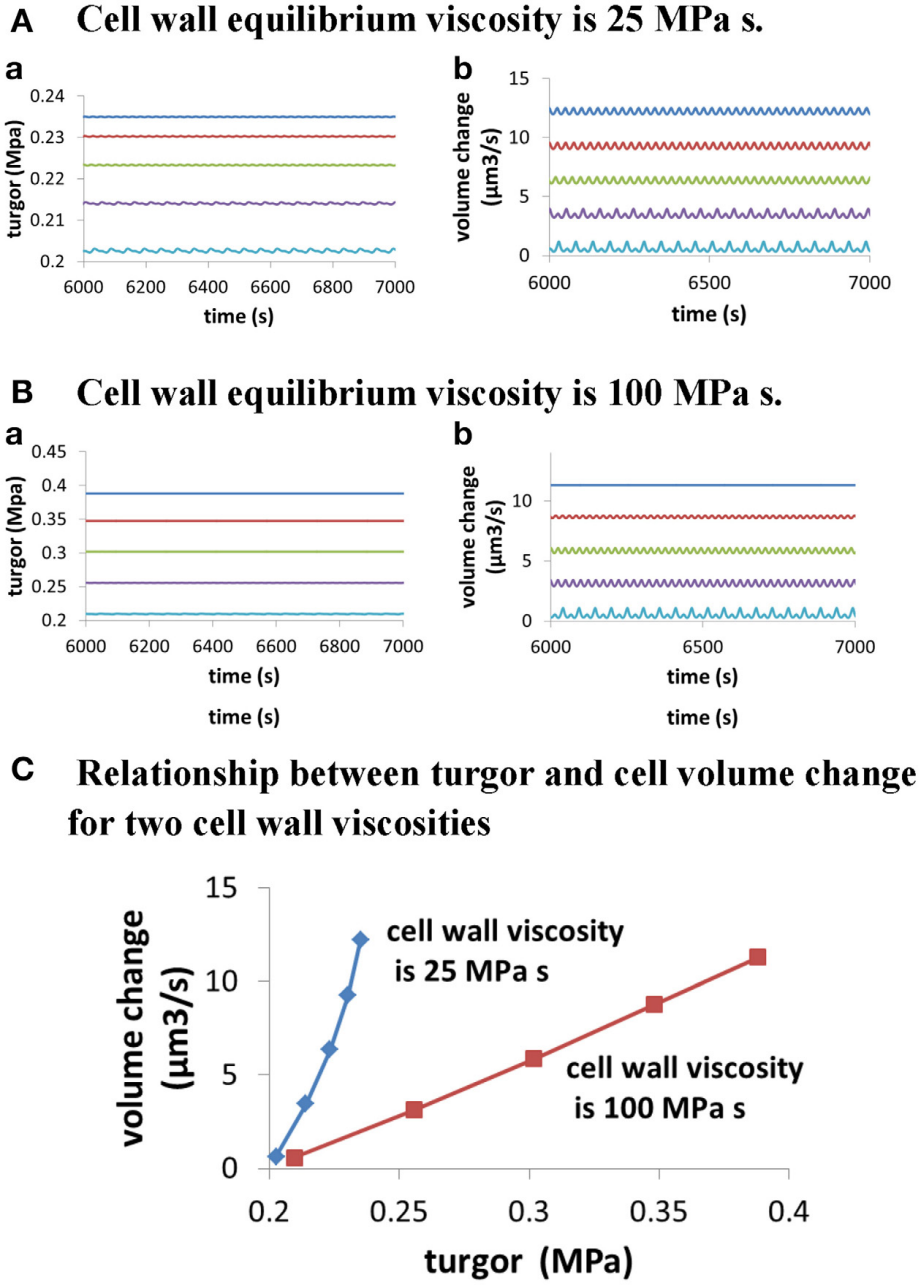
**Dependence of cellular turgor and volume change of pollen tube on osmolarity in the media for two cell wall viscosities**. In both **(A,B)**, osmolarity in the media increases from 0.06 to 0.46 Osm by increasing 0.1 Osm each time from top to bottom. When osmolarity increases in the media, cellular turgor decreases. **(A)** Dependence of cellular turgor and volume change of pollen tube for osmolarity in the media for low cell wall viscosity (25 MPa s) (i.e., large cell wall extensibility). **(B)** Dependence of cellular turgor and volume change of pollen tube on osmolarity in the media for high cell wall viscosity (100 MPa s). **(C)** Dependence of volume change of pollen tube on turgor for two cell wall viscosities.

**Figure 4:**
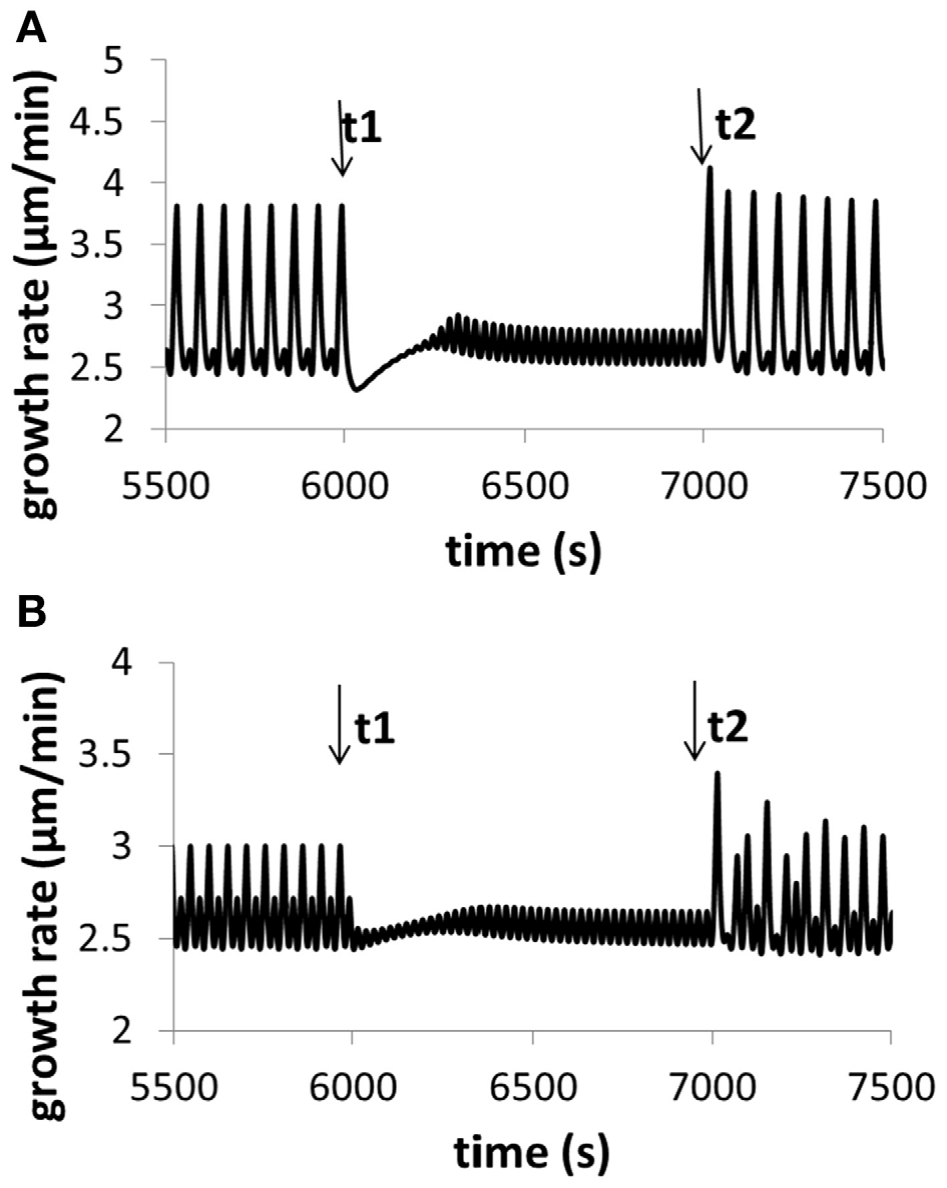
**Variation of growth rate for (A) different calcium concentrations in the media, and (B) different *p*H in the media**. Osmolarity in the media is 0.36O sm. **(A)** At time t1, [Ca^2+^] in the media increases from 0.13 to 1.3 mM (the reference value used in this work is 1 mM). The oscillatory amplitude of growth rate decreases and the baseline growth rate increases. At time t2, [Ca^2+^] returns the original 0.13 mM. The oscillatory amplitude of growth rate increases and the baseline growth rate decreases. **(B)** At time t1, *p*H in the media decreases to 5.1 from 5.7 (the reference value used in this work is 5.7). The oscillatory amplitude of growth rate decreases and the baseline growth rate slightly increases. At time t2, *p*H returns the original 5.7. The oscillatory amplitude of growth rate increases and the baseline growth rate slightly decreases. These results are qualitatively in agreement with experimental observations (Messerli and Robinson, 2003), although the range of varying *p*H in experiments (Messerli and Robinson, 2003) is much wider. Modeling results show that, when [Ca^2+^] in the media increases from 0.13 to 1.3 mM, although oscillatory amplitudes of growth rate change, the average growth rates for the two media conditions are approximately the same. The reason is as follows. The 10-fold increase (from 0.13 to 1.3 mM) of [Ca^2+^] in the media only leads to a change of 0.04 and 0.4% in the osmolarity in the media. Thus, the contribution of this 10-fold of [Ca^2+^] increase to the osmolarity in the media is insignificant.

**Figure 5:**
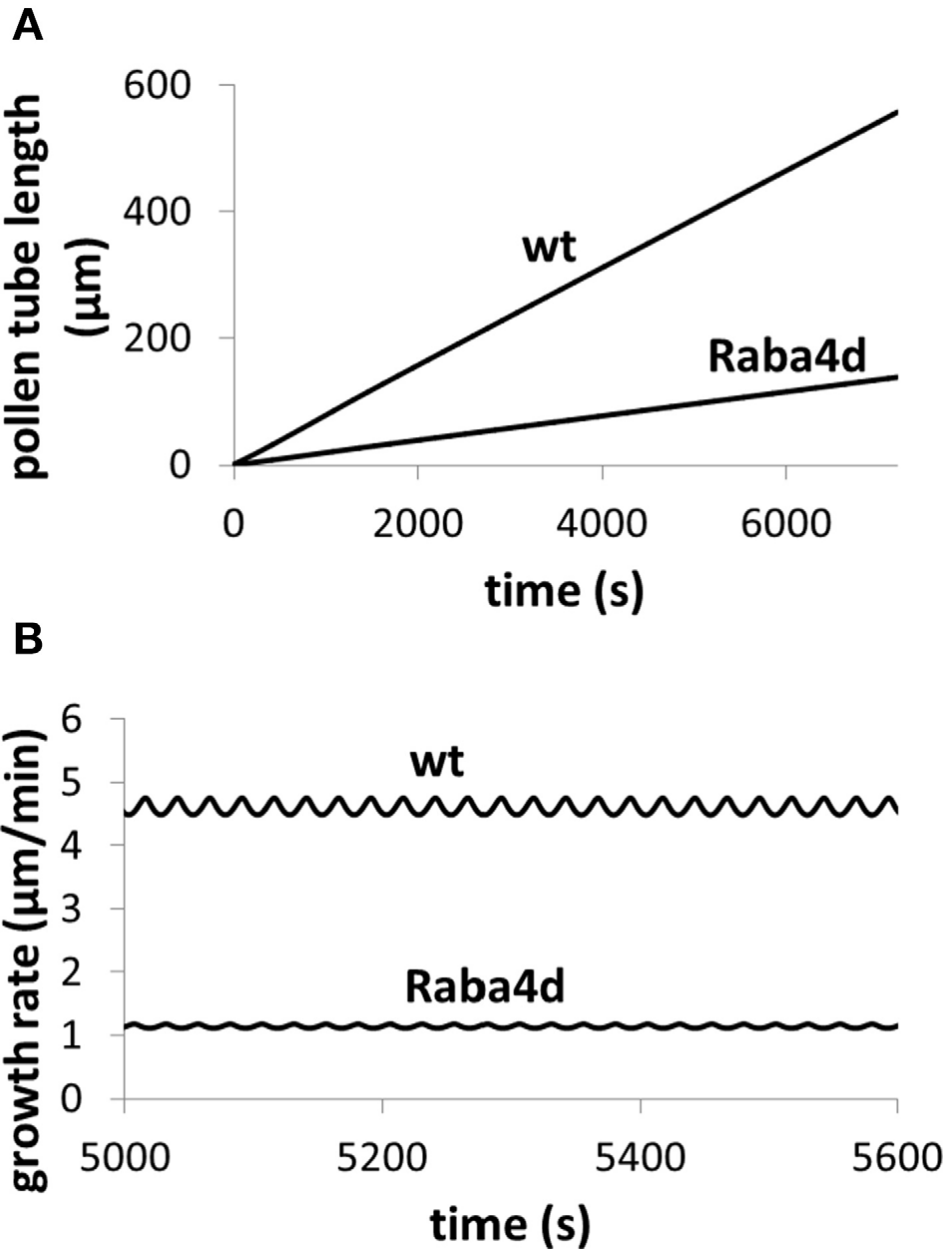
**Knockout of Raba4d, a member of the Rab family of small GTPase proteins, decreases pollen tube length**. Osmolarity in the media is 0.36 Osm. Fusion rate parameter is reduced from 21.5 μm^3^/(mM s) in wild type (wt) to 0.215 μm^3^/(mM s) in *Raba4d* mutant, mimicking that Raba4d regulates membrane trafficking. After knockout of Raba4d, membrane trafficking rate decreases. **(A)** Qualitatively reproduces experimental observations (Szumlanski and Nielsen, 2009): in *Raba4d* mutant, pollen tube length is shorter than that in wild type (Szumlanski and Nielsen, 2009). Following experimental observations (Szumlanski and Nielsen, 2009), the pollen tube radius is approximately 6 and 12 μm for wt and *Raba4d* respectively. Our simulations use the corresponding radius respectively. **(B)** Analyses pollen tube growth rate in both wild type and *Raba4d*.

Moreover, modeling predicts the following. (1) Change in turgor is very small when extracellular osmolarity changes and increasing extracellular osmolarity only slightly decreases cellular turgor if cell wall viscosity is low (i.e., cell wall extensibility is large). Moreover, for any fixed osmolarity in the media, oscillations in turgor are experimentally undetectable (<0.0009 MPa) (Figure 3). Experimentally, no oscillatory changes in turgor were observed within a resolution limit of ca. 0.005 MPa (Benkert et al., 1997). (2) Increasing extracellular calcium concentration or decreasing *p*H decreases growth oscillatory amplitude and increases baseline growth rate, respectively (Messerli and Robinson, 2003) (Figure 4). (3) Knockout of Raba4d decreases the average length of pollen tubes as measured after germination for 24 h *in vitro* (Szumlanski and Nielsen, 2009) (Figure 5). These modeling results are qualitatively in agreement with experimental observations.

### Role of turgor, osmotic pressure, cell wall extensibility, ion dynamics and hydrodynamics in pollen tube growth

Many experiments have shown that the growth rate and/or oscillatory dynamics depends on the osmolarity (Messerli and Robinson, 2003; Zonia and Munnik, 2004, 2007, 2011; Zonia et al., 2006; Kroeger et al., 2011). An osmotic zone for water flow at the pollen tube tip has been observed (Hill et al., 2012). Turgor is shown to be relatively stable during pollen tube growth (Benkert et al., 1997). Injection of oil changes turgor (Benkert et al., 1997). Moreover, changes in the ion concentration (e.g., calcium and *p*H) in the media are known to change oscillatory amplitude and baseline of pollen tube growth (Messerli and Robinson, 2003). Similarly, knockout of certain genes may also change growth rate. For example, knockout of Raba4d, a member of the Rab family of small GTPase proteins involved in vesicle transport, decreases the average length of pollen tubes as measured after germination for 24 h *in vitro* (Szumlanski and Nielsen, 2009).

Although these and other experimental observations indicate that pollen tube growth is regulated by hydrodynamics, cell wall and ion dynamics, little is known about the roles of these properties in pollen tube growth. For example, when osmolarity in the media is reduced, the oscillatory dynamics changes (Messerli and Robinson, 2003; Zonia and Munnik, 2004, 2007, 2011; Zonia et al., 2006; Kroeger et al., 2011). The questions we address here relate to the roles of hydrodynamics, cell wall and ion dynamics in the changes in oscillatory dynamics and growth rate.

**Figure 6:**
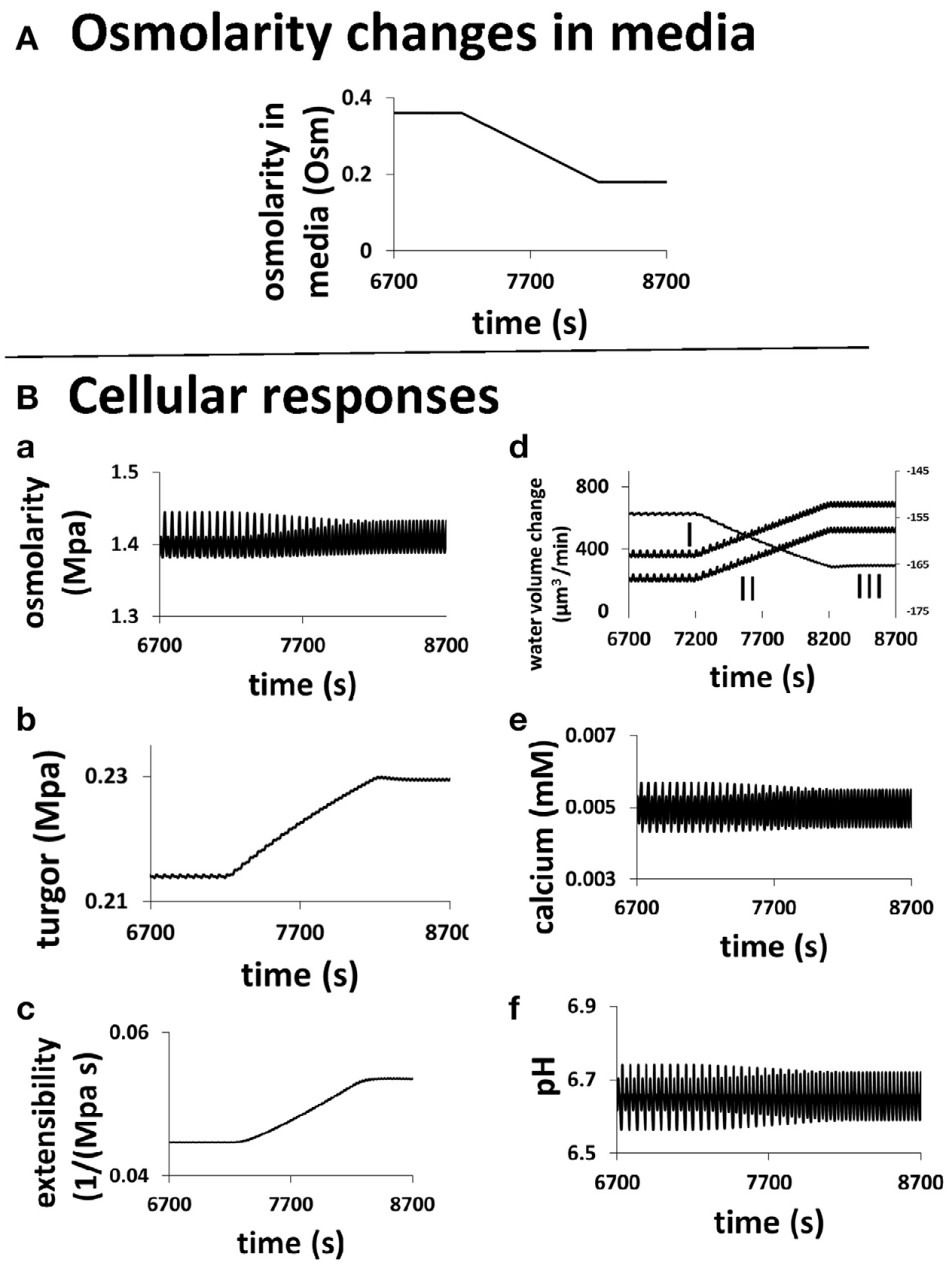
**Role of hydrodynamics, cell wall and ion dynamics in pollen tube growth. (A)** describes the transient change of osmolarity in the media. **(B)** is the cellular responses of a pollen tube to this change. Oscillatory periods in all properties decrease when osmolarity in the media linearly decreases from 0.36 to 0.18 Osm. **(a)** Cellular osmotic pressure remains relative unchanged; **(b)** turgor increases by ca.7%; **(c)** cell wall extensibility increases by ca. 20%; **(d)** (I) Water flow from the media to the cell by osmosis increases (Left y-axis). (II) The net water flow from the media to the cell increases (Left y-axis). (III) Water flow from the cell to the media by turgor increases (Right y-axis). **(e)** Cellular calcium concentration remains relatively unchanged. **(f)** Cellular *p*H remains relatively unchanged.

The underlying changes in the cell when the osmolarity in the media changes are shown in Figure 6. A pollen tube grows in a media of 0.36 Osm. At a specific time (here 7200 s), the osmolarity of the media starts to decrease linearly, after 1000 s it reaches 0.18 Osm (Figure 6). During this transient change, the average cellular osmotic pressure remains about the same and the cellular turgor increases only slightly from ca.0.214 to ca.0.229 MPa, but their dynamics change with the oscillatory period becoming shorter and the amplitude smaller (Figures 6A,B). The oscillatory amplitude of turgor is very small. In the media of 0.36 and 0.18 Osm, the oscillatory amplitude of turgor is ca. 0.0006 and ca. 0.0002 MPa, respectively. Experimentally, no oscillatory changes in turgor were observed within a resolution limit of ca. 0.005 MPa (Benkert et al., 1997). Therefore, our modeling analysis reveals that the oscillatory amplitude is well below the experimental resolution limit. Cell wall extensibility increases from ca. 0.045 to 0.054 MPa^−1^ s^−1^ (Figure 6C). During this change, water flow from the media to the cell increases by osmosis (Figure 6D). This is due mainly to the reduction of osmolarity in the media as cellular osmotic pressure remains about the same (Figure 6A). However, the increase in turgor in the pollen tube forces more water from the cell into the media. Because the increase in the water flow rate from the media to the cell through osmosis is larger than that due to turgor, the net water flow rate from the media to the cell increases. Furthermore, both the increase in the cell wall extensibility (Figure 6C) and the increase in turgor (Figure 6B) drive the increase in cell chamber volume. The increase in cell chamber volume is the same as the increase in water volume (Ortega, 1994, 2010), and therefore the volume of pollen tube increases. If radius of pollen tube does not change, increasing volume increases growth rate. However, if radius of pollen tube changes, conversion of volume change into growth rate should also include the effects of the change in radius (Figure 2) and the relationship between volume change and growth rate is included in Materials and Methods Section. In the following, we use volume change as a generic term for growth rate, as volume change is independent of the change in radius and it can be directly calculated based on biophysical principle (below).

As discussed by Hill et al. (2012), turgor plays two opposite roles in pollen tube growth: affecting the water entry and the expansion of cell wall chamber at the tip area (Hill et al., 2012). Our Figure 6 confirms the two opposite roles of turgor. First, a larger turgor forces more water out of the cell into the media, therefore this activity inhibits pollen tube growth. This is because the relationship between turgor and water flow follows Equation (1) based on the hydrodynamics of water flow in a pollen tube (Ortega, 1994, 2010).

(1) $$\frac{dv_{w}}{v_{w}dt}=\frac{L_{p}A_{osm}}{v_{w}}((\pi_{i}-\pi_{o})-P)$$

where *v*_*w*_ is the water volume in a pollen tube, *L*_*p*_ is the membrane hydraulic conductivity, *A*_*osm*_ is the membrane area allowing water permeable at the tip of pollen tube, π_*i*_ is the osmotic pressure in pollen tube, π_*o*_ is the osmotic pressure in the media, and *P* is the turgor pressure (relative to the pressure in the media). Furthermore, Hill et al. (2012) have shown experimentally that there is an osmotic zone at the pollen tube tip. Equation (1) is further discussed in Materials and Methods Section.

Secondly, a larger turgor increases the expansion of the cell wall chamber. Therefore, this role for turgor is in promoting pollen tube growth. This is because the relationship between turgor and the change of the cell wall chamber volume follows Equation (2) based on the Lockhart model (Ortega, 1994, 2010).

(2) $$\frac{dv_{c}}{v_{c}dt}=\frac{v_{ext}\Phi }{v_{c}}(P-P_{c})+\frac{1}{\varepsilon }\frac{dP}{dt}$$

Where *v*_*c*_ is the cell wall chamber volume, *v*_*ext*_ is the tip region of cell wall chamber volume that is extensible at the tip area, ϕ is the cell wall extensibility, *P*_*c*_ is the critical turgor pressure, and ε is the volumetric elastic modulus. Equation (2) is further discussed in Materials and Methods Section.

During pollen tube growth, the volume encompassed by the cell wall and the volume of cellular solution in a pollen tube are the same. For the simplicity of notation, in the following we use symbol *v* to denote both the water volume, *v*_*w*_, and the cell wall chamber volume, *v*_*c*_.

Furthermore, turgor in pollen tube is generated because the relative change in the volume of water Equation (1) and the volume of the cell wall chamber Equation (2) is equal during pollen tube growth (Ortega, 1994, 2010). Thus, turgor is a property regulated by pollen tube itself. Further analysis on the relationship between turgor, wall extensibility and pollen tube volume at different osmolarity in the media (Figure 3) shows that, when cell wall viscosity is low (25 MPa s) (i.e., cell wall extensibility is large), a small change in turgor (ca.16%; from ca. 0.203 to ca. 0.235 MPa) can cause a large-fold change in pollen tube volume change (ca.19-folds; from ca. 0.65 to ca. 12.21 μm^3^/s). However, when cell wall viscosity is high (100 MPa s), a relatively large change (ca. 85%; from ca. 0.210 to 0.388 MPa) is needed to cause a large-fold change in pollen tube volume change (ca. 20-folds, from 0.57 to 11.31 μm^3^/s). Our analysis also shows that, when cell wall viscosity is low, the volume change of pollen tube is not directly proportional to the turgor pressure. In addition, when cell wall viscosity is high, the volume change of pollen tube is more proportionally correlated to the turgor pressure (Figure 3). Therefore, as the pollen tube integrates hydrodynamics, cell wall and ion dynamics, it may sustain growth at different volume changes and simultaneously maintain relatively stable turgor when cell wall extensibility is large. Experimentally, Benkert et al. (1997) has shown that turgor is stable during pollen tube growth. Additionally, despite the fact that changing osmolarity may sustain growth at different volume changes, the average cellular calcium concentration and the *p*H remain about the same (Holdaway-Clarke and Hepler, 2003), as observed by modeling in Figures 6E,F.

Turgor plays two roles, affecting water flow and expansion of cell chamber volume (Hill et al., 2012). Therefore, hydrodynamics is closely associated with cell wall chamber volume in pollen tube growth. In this context we ask what the roles of turgor and the cell wall are in pollen tube growth if a molecular event (e.g., knocking out an essential gene) perturbs the cell wall extensibility?

**Figure 7:**
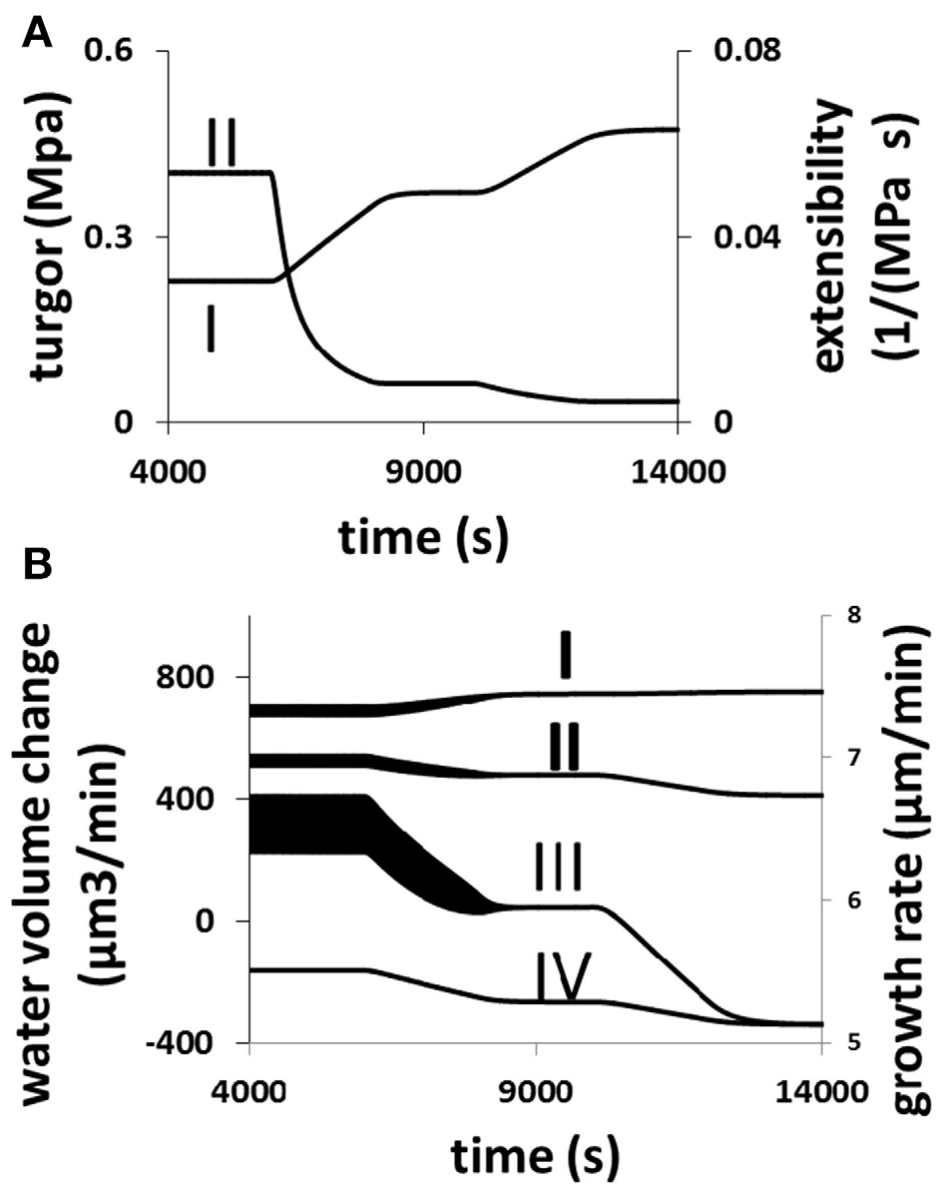
**Responses of a growing pollen tube to the change in the viscosity of cell wall**. Osmolarity in the media is 0.18 Osm. Equilibrium viscosity, a parameter that affects the viscosity of cell wall Equation (6), changes as follows. *t*1 = 6000 s, *t*2 = 8000 s, *t*3 = 10,000 s, and *t*4 = 12000 s. Before t1: 25 MPa s; between t1 and t2, linear increase from 25 to 125 MPa s; between t2 and t3, 125 MPa s; Between t3 and t4, linear increase from 125 to 225 MPa s; after t4: 225 MPa s. **(A,B)** Show the cellular responses. **(A)** Turgor increases (I) and cell wall extensibility (II) (cell wall extensibility is the inverse of the viscosity) decreases simultaneously. **(B)** Water flow from the media to the cell by osmosis (I) increases, and water flow from the cell to the media by turgor (IV) also increases. The overall result is that the net water flow from the media to the cell (II) decreases and therefore growth rate (III) also decreases. The wide black bands in this figure indicate the oscillations. The width of each band represents the oscillatory amplitude.

The response of a growing pollen tube to the changes in cell wall extensibility as a result of varying the equilibrium viscosity of cell wall materials [Equation (6) in Materials and Methods Section] is shown in Figure 7. When cell wall extensibility decreases, the pollen tube builds up its turgor (Figure 7A). But as the turgor increases, the growth rate is low (Figure 7B) due to cell wall extensibility being low (Figure 7A). Whilst the increase in turgor helps to increase the volume of the cell wall chamber, the decrease in the extensibility of the cell wall restricts cell wall expansion Equation (2). Furthermore, when cell wall extensibility decreases, cellular osmotic pressure increases (Figure 7B). This increases water flow from the media into the pollen tube (Figure 7B). However, the increase in turgor forces water out of the cell at a higher rate (Figure 7B). Therefore, net volume change of pollen tube decreases (Figure 7B). Thus, growth rate decreases when cell wall extensibility decreases.

**Figure 8:**
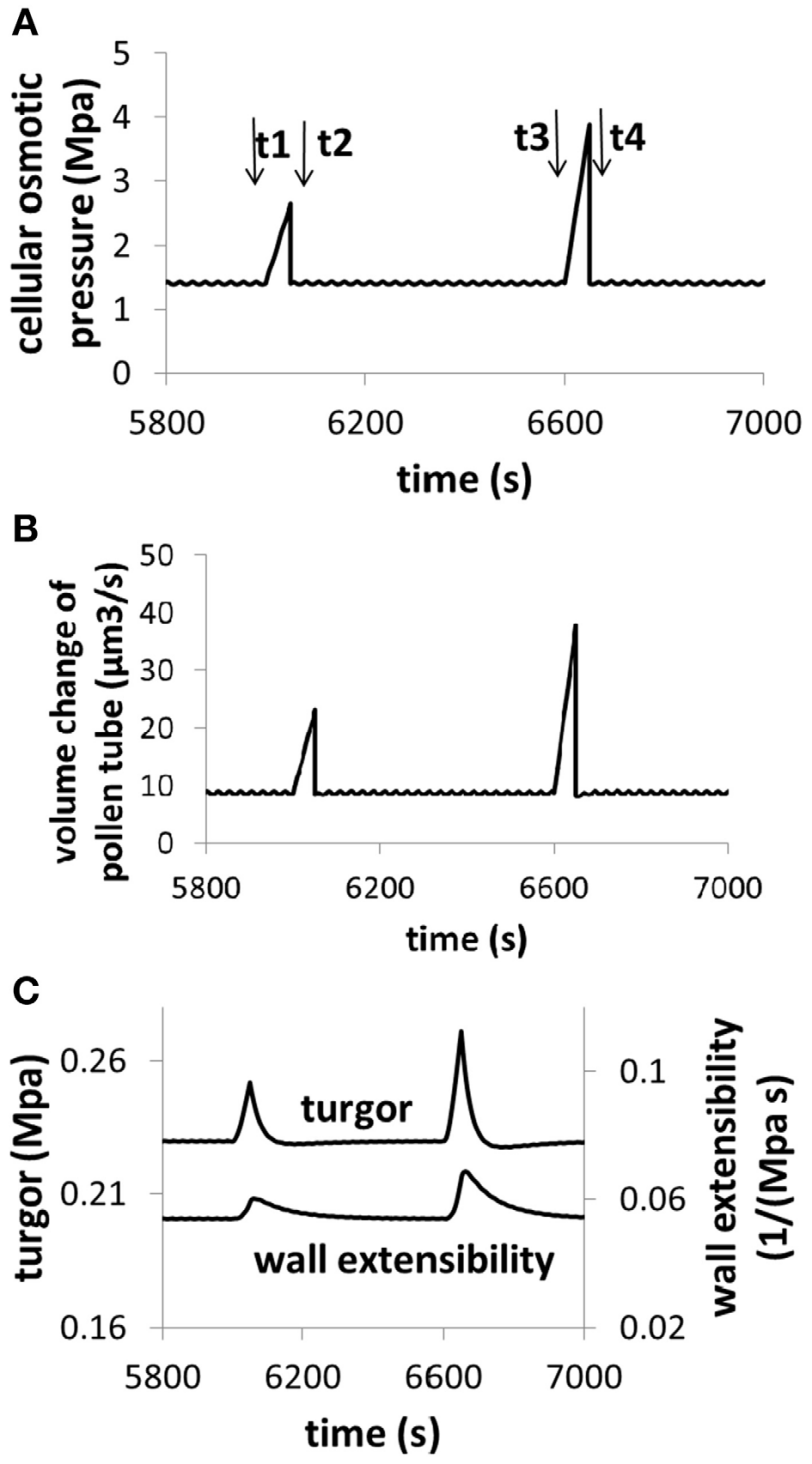
**Dependence of volume change, turgor, and cell wall extensibility on cellular osmotic pressure**. Osmolarity in the media is 0.36 Osm. **(A)** An example for increasing cellular osmolarity (e.g., by injection of oil). Before time t1, the cellular osmolarity is 0.5 M. From t1 to t2, cellular osmolarity linearly increases from 0.5 to 0.75 M due to injection of oil. At time t2, the cellular osmolarity is reset to be 0.5 M. From t3 to t4, cellular osmolarity linearly increases from 0.5 to 1.0 M due to injection of oil. At time t4, the cellular osmolarity is reset to be 0.5 M. **(B)** Dependence of volume change on cellular osmotic pressure. **(C)** Dependence of turgor and cell wall extensibility on cellular osmotic pressure. The modeling result of turgor response following the increase of cellular osmotic pressure (8c) is qualitatively in agreement with experimental observations in which cellular osmotic pressure is increased by injection of oil (Benkert et al., 1997).

The analysis described by Figures 6, 7 reveals an important feature of pollen tube growth and, that is, the role of hydrodynamics and cell wall in pollen tube growth are context dependent. When osmolarity in the media decreases, turgor increases, leading to an increase in pollen tube growth rate. However, when cell wall extensibility decreases, turgor also increases, leading to a decrease in the volume change of pollen tube. Therefore, if we consider only turgor, increasing turgor may increase or decrease the volume change, depending on the context of pollen tube growth. Further analysis reveals that if cellular osmolarity increases (e.g., by injection of oil, Benkert et al., 1997), both turgor and cell wall extensibility increase, leading to an increase in volume change. In this context, increasing turgor increases volume change of pollen tube (Figure 8).

Our analysis reveals that when pollen tube growth is perturbed, hydrodynamics, cell wall and ion dynamics work together to change pollen tube volume (Figures 6, 7). Moreover, their roles in pollen tube growth are context dependent. Therefore, pollen tube growth is a process regulated by an integrated network of processes that involve hydrodynamics, cell wall and ion dynamics. In the next section we develop the methodology to identify the main regulatory points in pollen tube growth.

### Dissecting the regulation of pollen tube growth

We adapt regulation analysis originally developed for metabolic networks (Rossell et al., 2006; Haanstra et al., 2008) to dissect the regulation of pollen tube growth based on the concept of regulation coefficients (Rossell et al., 2006; Haanstra et al., 2008).

Regulation coefficients are strictly defined as follows. When pollen tube growth is perturbed (e.g., by changing osmolarity in the media), pollen tube volume changes. Regulation coefficients quantify the contribution of all properties involving in pollen tube growth (osmotic pressure, turgor, and cell wall extensibility) to the volume change of pollen tube. We note that, following the original definition (Rossell et al., 2006; Haanstra et al., 2008), regulation coefficients just compare two pollen tube growth states at two different conditions. For example, pollen tube growth in the media of 0.36 Osm is compared to pollen tube growth in the media of 0.18 Osm. Therefore, calculation of regulation coefficients is based solely on the fact that the relative change in the volume of water and the volume of the cell wall chamber is equal at any condition of pollen tube growth (Ortega, 1994, 2010). Moreover, regulation coefficients do not include information about how the oscillatory states in the two media conditions are established, and in particular they do not study which events occur first. For example, when pollen tube grows, cell wall extensibility may change first (Winship et al., 2010), and water flow from media to the cell then follows. This process leads to that the relative change in the volume of water and the volume of the cell wall chamber is equal when the oscillatory growth state is established. Regulation coefficients only consider these equal volumes and do not study how cell wall extensibility changes first.

In Figure 6 we show the response of a pollen tube to changes in the osmolarity of the media; decreasing from 0.36 to 0.18 Osm. For both cases, the pollen tube shows oscillatory growth, albeit with different periods and amplitudes (Figure 6). In order to identify the main regulation points, we derive regulation coefficients as follows and in the Materials and Methods Section.

For osmolarity in the media to be 0.36 Osm, ${\bar{\left(\frac{d\nu }{dt}\right)}}_{O1}$; ${\left(\bar{L_{p}A_{osm}\pi_{i}}\right)}_{O1}$, ${\left(\bar{L_{p}A_{osm}\pi_{o}}\right)}_{O1}$ and $\bar{{\left(L_{p}A_{osm}P\right)}_{O1}}$ are the average of the left hand of, and each term of the right hand of Equation (1) over an exact period. For a simple periodic oscillatory shape such as the oscillations in Figure 2A, an exact period starts from the time when a maximum of volume change has reached to the time when the next maximum has reached. For the oscillations with a regular shape with a large peak and a small peak such as the oscillations in Figures 2B,C, an exact period starts from the time when a maximum of a large peak has reached to the time when the maximum of the next large peak has reached. For osmolarity in the media to be 0.18 Osm, ${\bar{\left(\frac{d\nu }{dt}\right)}}_{O2}$; ${\left(\bar{L_{p}A_{osm}\pi_{i}}\right)}_{O2}$, ${\left(\bar{L_{p}A_{osm}\pi_{o}}\right)}_{O1}$ and $\bar{{\left(L_{p}A_{osm}P\right)}_{O2}}$ are the respective average. According to Equation (1), we have *R*_π_*i*__ + *R*_π_*o*__ + *R*_*P*_ = 1, with $R_{\pi_{i}}^{w}=\frac{{\left(\bar{L_{p}A_{osm}\pi_{i}}\right)}_{O1}-{\left(\bar{L_{p}A_{osm}\pi_{i}}\right)}_{O2}}{{\bar{\left(\frac{d\nu }{dt}\right)}}_{O1}-{\bar{\left(\frac{d\nu }{dt}\right)}}_{O2}}$ ; $R_{\pi_{0}}^{w}=-\frac{{\left(\bar{L_{p}A_{osm}\pi_{o}}\right)}_{O1}-{\left(\bar{L_{p}A_{osm}\pi_{o}}\right)}_{O2}}{{\bar{\left(\frac{d\nu }{dt}\right)}}_{O1}-{\bar{\left(\frac{d\nu }{dt}\right)}}_{O2}}$; and $R_{P}^{w}=-\frac{{\bar{\left(L_{p}A_{osm}P\right)}}_{O1}-{\bar{\left(L_{p}A_{osm}P\right)}}_{O2}}{{\bar{\left(\frac{d\nu }{dt}\right)}}_{O1}-{\bar{\left(\frac{d\nu }{dt}\right)}}_{O2}}$. The *R*^*w*^_π_*i*__ ; *R*^*w*^_π_0__ and *R*^*w*^_*P*_ are the regulation coefficients of cellular osmotic pressure, osmotic pressure in the media; and turgor pressure for water volume change in the pollen tube, respectively. Following the same principle, the regulation coefficients of turgor pressure (*R*^*c*^_*P*_), cell wall extensibility (*R*^*c*^_Φ_) and temporal change in turgor pressure (*R*^*c*^_*o*_) for change in wall chamber volume can also be derived (Materials and Methods Section).

Since the volume encompassed by the cell wall and the volume of cellular solution in a pollen tube are the same, these regulation coefficients quantitatively measure the regulation strength of each property on pollen tube growth by calculating the contribution of change in each property to the volume change in pollen tube. For example, when the growth state in the media of 0.36 Osm is compared to that in the media of 0.18 Osm (Figure 6), *R*^*w*^_*P*_ > 0 (*R*^*w*^_*P*_ < 0) implies that the change in turgor pressure makes positive (negative) contribution to the volume change of pollen tube, respectively. Thus, turgor pressure positively (negatively) regulates water volume change in the pollen tube, respectively.

Calculation of regulation coefficients reveals that, when osmolarity in the media changes from 0.36 to 0.18 Osm (Figure 6), the regulation coefficients for the change in volume change are: *R*^*w*^_π_*i*__ = 0.004, *R*^*w*^_π_*o*__ = 1.030, *R*^*w*^_*P*_ = −0.034, *R*^*c*^_*P*_ = 0.789, *R*^Φ^_*P*_ = 0.214 and *R*^*c*^_*o*_ = −0.003. These regulation coefficients reveal that the main regulatory points are the extracellular osmolarity for water flow and the turgor for cell wall chamber volume. The positive and negative values for the two turgor regulation coefficients mean that turgor plays two roles in pollen tube growth and that the two roles oppose each other to regulate pollen tube growth. These two opposite roles were also discussed in literature (Hill et al., 2012). However, when equilibrium cell wall viscosity changes from 125 to 225 MPa s (this change leads to changes in cell wall extensibility), the regulation coefficients for the change in growth rate are: *R*^*w*^_π_*i*__ = −0.097, *R*^*w*^_π_*o*__ = 0.000, *R*^*w*^_*P*_ = 1.097, *R*^*c*^_*P*_ = −3.084, *R*^Φ^_*P*_ = 4.087 and *R*^*c*^_*o*_ = −0.003. *R*^*w*^_π_*o*__ is a very small negative number. With the accuracy of three decimal digits, it is approximately zero. These regulation coefficients reveal that the main regulatory points are turgor for water flow and cell wall extensibility for cell wall chamber volume. In conclusion the change in the main regulatory points (Figures 6, 7) reflects the context-dependent regulation of pollen tube growth.

## Discussion

Experimental information accumulated for many years indicates that hydrodynamics, cell wall and ion dynamics are important properties regulating pollen tube growth. However, the underlying regulatory mechanism is unclear. This is mainly because of the lack of methodology integrating these properties (Liu and Hussey, 2011; Kroeger and Geitmann, 2012). In this work, using a variety of experimental information, our integrative model develops insights into how turgor, cell wall extensibility, osmolarity in the media and in the cell, and ions play their roles in pollen tube growth. Specifically, intrinsic coupling of hydrodynamics, cell wall and ion dynamics reproduces key experimental observations. That is: (1) that the hypertonic condition leads to a much longer oscillatory period and that the hypotonic condition halves the oscillatory period; (2) that oscillations in turgor are experimentally undetectable; (3) that increasing the extracellular calcium concentration or decreasing the *p*H decreases the growth oscillatory amplitude; (4) that knockout of Raba4d, a member of the Rab family of small GTPase proteins, decreases pollen tube length after germination for 24 h. The model generated here reveals that (1) when cell wall extensibility is large, pollen tube may sustain growth at different volume changes and maintain relatively stable turgor; (2) turgor increases if cell wall extensibility decreases; (3) increasing turgor due to decrease in osmolarity in the media, although very small, increases volume change. However, increasing turgor due to decrease in cell wall extensibility decreases volume change. In this way regulation of pollen tube growth by turgor is context dependent. By changing the osmolarity in the media, the main regulatory points are extracellular osmolarity for water flow and turgor for wall chamber volume. However, if the viscosity of cell wall changes, the main regulatory points are turgor for water flow and wall extensibility for wall chamber volume.

Currently, there are two main models of pollen tube growth. The cell wall model considers that cell wall mechanical properties control growth (Winship et al., 2010, 2011) and the hydrodynamic model suggests the intracellular pressure, turgor, controls growth (Zonia and Munnik, 2011). The outcomes of these two main pollen tube growth models do not appear to be reconcilable (Winship et al., 2010, 2011; Zonia and Munnik, 2011). In particular, the role of the turgor pressure is subjected to intense debate (Winship et al., 2010, 2011; Zonia and Munnik, 2011). Our modeling analysis reveals that (1) pollen tube may sustain growth at different volume changes and maintain relatively stable turgor when cell wall extensibility is large; (2) turgor increases if cell wall extensibility decreases; (3) increasing turgor may increase or decrease growth rate depending on how pollen tube growth is perturbed. In this way regulation of pollen tube growth is context dependent. Our work reveals that to explain the experimental observations pollen tube growth has to be considered to be context dependent. In particular a dual role of turgor in water flow and cell wall chamber has to be taken into account, as previously discussed in the literature (Hill et al., 2012).

As the pollen tube integrates hydrodynamics, cell wall and ion dynamics, there is a complex relationship among turgor, cell wall extensibility, pollen tube volume change and osmolarity in the media (Figures 3, 6, 7). First, when cell wall viscosity is small (i.e., cell wall extensibility is large), a relatively stable turgor may sustain growth at different volume changes. However, when cell wall viscosity is large, a relatively large change in turgor is needed to cause a large-fold change in pollen tube volume. Second, the volume change of pollen tube is not directly proportional to the turgor pressure if cell wall viscosity is small. In addition, when cell wall viscosity is large, the volume change of pollen tube is more proportionally correlated to the turgor pressure. Therefore, the dependence of volume change of pollen tube on turgor depends also on cell wall viscosity. Third, the same change in osmolarity in the media may lead to different relationships between volume change and turgor, depending on cell wall viscosities. For example, as shown in Figure 3, when osmolarity in the media decreases from 0.36 to 0.16 Osm, 0.016 MPa increase in turgor (from 0.214 to 0.230 MPa) corresponds to a 2.69-fold increase in volume change of pollen tube (from 3.45 to 9.27μm^3^/s) if cell wall viscosity is 25 MPa s. However, for the same osmolarity change, 0.093 MPa increase in turgor change (from 0.255 to 0.348 MPa) corresponds to a 2.78-fold change in volume change of pollen tube (from 3.13 to 8.71 μm^3^/s) if cell wall viscosity is 100 MPa s. We note that, if the radius does not change, volume change and growth rate are equivalent.

During the model development in this work, we have used experimental information available for different pollen species. Therefore, our model development has assumed that pollen tube growth for different species follows the same principle as described in Figure 1. In addition, although this work has attempted to use a wide range of experimental information available in the literature, many important aspects of pollen tube growth, such as actin cytoskeleton organization, fertilization and self-incompatibility and intracellular signaling molecules, have not been included in the current model. Therefore, although we consider that our model developed in this work is an effort for analysing pollen tube growth as an integrative system, we note some noticeable differences between our model outcomes and experimental observations. Oscillations in growth rate in experimental observations (Messerli and Robinson, 2003; Zonia and Munnik, 2004, 2007, 2011; Zonia et al., 2006; Kroeger et al., 2011) usually have irregular shapes. In addition, secondary frequency in the oscillations of pollen tube growth was also reported (Michard et al., 2008). Our model always generates oscillations with a regular shape. Moreover, our model does not generate secondary frequency as observed in Michard et al. (2008). In addition, based on experimental observation that shows the tip of the pollen tube is significantly softer than the shank region (Zerzour et al., 2009), our model considers that cell wall extensibility occurs at the tip during pollen tube growth. An interesting recent paper has shown that stiffness along the pollen tube can be explained by its geometry (Vogler et al., 2013): this result may indicate that the mechanical cell wall properties for the entire pollen tube could be uniform. Thus, modeling the irregular shapes, secondary frequencies and effects of different cell wall properties are among the future challenges of integrative model developments in pollen tube growth.

## Materials and methods

### Model and experimental data

The model developed here intrinsically couples hydrodynamics, cell wall and ion dynamics following kinetic and thermodynamic principles. The model describes an integrated system that governs pollen tube growth and that using this system the pollen tube itself can adjust growth dependent on the nature and the perturbations of the growth conditions. The main experimental observations used for model development are included in SI-Appendix. Here we summarize the key points: (a) Oscillation: with reference to an oscillatory period of ca. 50 s in the isotonic condition, the hypertonic condition causes a longer oscillatory period (ca. 100 s) and the hypotonic condition causes a shorter oscillatory period (ca. 25 s) (Zonia and Munnik, 2004, 2007, 2011; Zonia et al., 2006). Qualitatively similar data are also in the literature (Messerli and Robinson, 2003; Kroeger et al., 2011). A change in the concentration of Ca^2+^ or of *p*H is capable of changing the oscillatory amplitude (Messerli and Robinson, 2003). (b) Growth rate: the growth rate of *in vitro*-grown an Arabidopsis pollen tube is 4.07 ± 0.36 and 4.5 ± 1.0 μm min^−1^ (Ketelaar et al., 2008; Szumlanski and Nielsen, 2009), respectively. (c) Turgor: ca. 0.1–ca. 0.4 MPa (Benkert et al., 1997). The injection of oil was shown to change the turgor (Benkert et al., 1997). No oscillatory changes in turgor were observed within a resolution limit of ca. 0.005 MPa. (d). Ion dynamics: both voltage-gated and stretch-activated transporters are linked with ion dynamics (Holdaway-Clarke and Hepler, 2003; Dutta and Robinson, 2004; Hepler et al., 2012). (e) Membrane trafficking: knockout of Raba4d, a member of the Rab family of small GTPase proteins, decreases the average length of pollen tubes as measured after germination for 24 h *in vitro* (Szumlanski and Nielsen, 2009). (f) Vesicle fusion: vesicle secretion is known to be related to cellular Ca^2+^ concentration (Roy et al., 1999; Blank et al., 2001). (g) Water flow: water flow is related to pollen tube growth (Zonia et al., 2006; Hill et al., 2012). (h) Cell wall: the thickness is ca. 0.2–ca. 0.5 μm (Lancelle and Hepler, 1988, 1992; Holdaway-Clarke and Hepler, 2003; McKenna et al., 2009). The outer radius of a pollen tube is ca. 5 μm in the media of 0.36 Osm (Zonia and Munnik, 2004).

### Equations

The equations used for modeling the intrinsic coupling of hydrodynamics, cell wall and ion dynamics in pollen tube and for dissecting the regulation of pollen tube growth are described below. As described by these equations, hydrodynamics, cell wall and ion dynamics in pollen tube are intrinsically coupled. When pollen tube growth is subject to any perturbation, pollen tube itself adjusts the three types of dynamics, leading to change in both growth dynamics and rate.

#### Volume change, length change (growth rate), and radius change in pollen tube

In this work, pollen tube is assumed to have a regular cylindrical shape. Its volume, length, area and outer radius are *v, L, A, r*, respectively. Therefore, *v* = π*r*^2^ *L* and *A* = 2π *rL*. Thus, when the volume of pollen tube is converted into growth rate (length change), the following equation is generally valid: $\frac{dv}{dt}=2\pi rL\frac{dr}{dt}+\pi r^{2}\frac{dL}{dt}$. This leads to $\frac{dL}{dt}=\frac{\frac{dv}{dt}-2\pi rL\frac{dr}{dt}}{\pi r^{2}}$. Growth rate in Figure 2 is calculated using this equation based on the volume change and radius change. If radius is fixed (not time-dependent), we have $\frac{dL}{dt}=\frac{\frac{dv}{dt}}{\pi r^{2}}$. All figures apart from Figure 2 are calculated using this equation.

#### Rate of change in water volume

The relative rate of change in water volume is described by Equation (1) as shown above. Hill et al. have analyzed that, if cell wall is permeable to water everywhere in the pollen tube, the pollen tube will grow exponentially with time (Hill et al., 2012). This is because the change in water volume of pollen tube is proportional to the volume itself ($\frac{A_{Osm}}{v}$ is a constant for a fixed radius). However, exponential growth of pollen tube has not been experimentally observed. Using experiments, Hill et al. have further shown that there is an osmotic zone in pollen tube tip (Hill et al., 2012). Therefore, *A*_*Osm*_ is approximately fixed during pollen tube growth. We consider that solute reflection coefficient (Ortega, 1994, 2010) is always 1 in this work and it has not been explicitly included in Equation (1). In addition, we do not consider water loss via transpiration (Ortega, 1994, 2010).

#### Rate of change in cell wall chamber volume

The relative rate of change in cell wall chamber is described by Equation (2) as shown above (Ortega, 1994, 2010). If cell wall extensibility of pollen tube is the same everywhere, Equation (2) becomes $\frac{dv}{vdt}=\phi (P-P_{c})+\frac{1}{\varepsilon }\frac{dP}{dt}$ (Ortega, 1994, 2010). Thus, the pollen tube will grow exponentially with time. This is because the change in cell wall chamber volume of the pollen tube is proportional to the volume itself. However, exponential growth of pollen tube has not been experimentally observed. Experimentally, Zerzour et al. have shown that the stiffness profile of a growing pollen tube as measured by micro-indentation reveals that the tip of the tube is significantly softer than the shank region (Zerzour et al., 2009). Therefore, cell wall extensibility of pollen tube is not the same everywhere. As the tip of the pollen tube is significantly softer than the shank region, we consider that pollen tube extension occurs only at the pollen tube tip region. Thus, at the pollen tube tip region with a volume of *v*_*ext*_, the cell wall extensibility is ϕ. At the pollen tube shank region, we assume that the cell wall extensibility is zero. When a pollen tube with a volume of *v* and the same cell wall extensibility of ϕ everywhere is compared to a pollen tube with a volume of *v* and wall extensibility of ϕ for the tip region of the volume (*v*_*ext*_) only (cell wall extensibility for the other part of pollen tube is zero), the overall cell wall extensibility of the whole pollen tube for the latter is reduced by a factor of $\frac{v_{ext}}{v}$. For the latter case, if the tip region volume (*v*_*ext*_) is reduced, as far as the growth of the whole pollen tube is concerned, the overall cell wall extensibility is reduced. If the tip region volume (*v*_*ext*_) is reduced to zero, pollen tube growth cannot occur anymore. In addition, by considering that pollen tube has a regular cylindrical shape, the volume ratio $\frac{v_{ext}}{v}$ is the same as the corresponding surface area ratio or the corresponding length ratio. Thus, Equation (2) has considered the case that pollen tube extension only occurs at the volume (or surface area) of tip region.

#### Cellular osmotic pressure

(3) $$\pi_{i}=RT({\left[Ca^{2+}\right]}_{i}+{\left[H^{+}\right]}_{i}+{\left[K^{+}\right]}_{i}+{\left[Cl^{-}\right]}_{i}+{\left[Osm\right]}_{i})$$

where R is gas constant, T is temperature. [*Ca*^2+^]_*i*_, [*H*^+^]_*i*_, [*K*^+^]_*i*_ and [*Cl*^−^]_*i*_ are cellular concentrations of these ions,and their values are determined by ion dynamics see below, Equation (10) due to the action of transporters. [*Osm*]_*i*_ is the concentration of other cellular molecules that contribute to osmolarity in cytosol. Hydrodynamics is coupled with ion dynamics in the cytosol via Equation (3).

#### Extracellular osmotic pressure

(4) $$\pi_{o}=RT({\left[Ca^{2+}\right]}_{o}+{\left[H^{+}\right]}_{o}+{\left[K^{+}\right]}_{o}+{\left[Cl^{-}\right]}_{o}+{\left[Osm\right]}_{o})$$

where R is gas constant, T is temperature. [*Ca*^2+^]_*o*_, [*H*^+^]_*o*_, [*K*^+^]_*o*_ and [*Cl*^−^]_*o*_ are concentrations of these ions in the media. Experimentally we can change these values. [*Osm*]_*o*_ is the concentration of other molecules that contribute to osmolarity in the media. Hydrodynamics is coupled with ion dynamics via Equation (4).

#### Rate of change in turgor pressure

Cell wall dynamics is coupled with hydrodynamics, as the relative change in the volume of water and cell wall chamber is equal during pollen tube growth. The result of this coupling is that the rate of change in turgor pressure is described by Equation (5) (Ortega, 1994, 2010).

(5) $$\frac{dP}{dt}=\varepsilon \text{​}\left(\frac{L_{P}A_{Osm}}{v}(\pi_{i}-\pi_{o}-P)-\frac{v_{ext}}{v}\phi (P-P_{C})\right)$$

#### Cell wall extensibility and viscosity of cell wall material

Cell wall extensibility, ϕ, is the inverse of the viscosity, η, which is calculated using Equation (6) (Kroeger et al., 2008, 2011).

(6) $$\frac{d\eta }{dt}=-\frac{\eta_{s}R_{s}}{h}+k_{1}(\eta_{eq}-\eta )$$

where *R*_*s*_ is vesicle secretion rate to cell wall, *h* is the cell wall thickness, and η_*eq*_ is the equilibrium viscosity, and *k*_1_ is a rate constant.

#### Vesicle secretion rate to cell wall

Vesicle secretion rate to cell wall, *R*_*s*_, is coupled with ion dynamics via [*Ca*^2+^]_*i*_ and it is described using Equations (7) and (8) (Kroeger et al., 2008, 2011).

(7) $$R_{s}=k_{2}{\left[Ca^{2+}\right]}_{i}$$

(8) $$k_{2}=\frac{k_{2a}}{r^{2}}$$

where *k*_2_ and *k*_2*a*_ are the parameters describing the dependence of vesicle secretion rate on cellular [*Ca*^2+^ ]_*i*_, which follows ion dynamics due to the action of transporters; *r* is the pollen tube outer radius. The unit of *k*_2_ and *k*_2*a*_ is m/M s and m^3^/M s, respectively. In this work, following Kroeger et al. (2008, 2011), we use *k*_2*a*_ = 2.15e−14 m^3^/M s. Thus, *k*_2_ is 4.1e−4 m/M s for a pollen tube radius of 5.0e−6 m. Ion dynamics (calcium dynamics) is coupled with vesicle secretion rate via Equation (7).

#### Rate of change in cell wall thickness

Rate of change in cell wall thickness is described using Equation (9) (Kroeger et al., 2008, 2011).

(9) $$\frac{dh}{dt}=-\frac{3(r^{2}-r_{i}^{2})}{2r^{2}}\frac{dL}{dt}+R_{s}$$

where *r* and *r*_*i*_ are the outer and inner radii, respectively. $\frac{dL}{dt}$ is the pollen tube growth rate. Rate of change in cell wall thickness is coupled with ion dynamics via the dependence of vesicle secretion rate on cellular [*Ca*^2+^ ]_*i*_ Equation (7).

#### Ion dynamics

Rate of change in the concentration of four cellular ions, [*Ca*^2+^]_*i*_, [*H*^+^]_*i*_, [*K*^+^]_*i*_ and [*Cl*^−^]_*i*_ are described using Equation (10) (voltage-gated transporters for pollen tube growth were previously studied in detail Liu et al., 2010).

(10) $$\begin{array}{l} \frac{d{\left[Ca^{2+}\right]}_{i}}{dt}=-\frac{OV(I_{vg3}+I_{sa1})}{2F}-k_{Ca^{2+}}({\left[Ca^{2+}\right]}_{i}-{\left[Ca^{2+}\right]}_{s})\\ \;-\frac{dv}{vdt}{\left[Ca^{2+}\right]}_{i}+Ca_{buffering}^{2+}\\ \;\frac{d{\left[H^{+}\right]}_{i}}{dt}=-\frac{OV(I_{vg4}+2I_{vg5})}{F}-k_{H^{+}}({\left[H^{+}\right]}_{i}-{\left[H^{+}\right]}_{s})\\ \;-\frac{dv}{vdt}{\left[H^{+}\right]}_{i}+H_{buffering}^{+}\\ \;\frac{d{\left[K^{+}\right]}_{i}}{dt}=-\frac{OV(I_{vg1}+I_{vg2}+I_{sa2})}{F}-k_{K^{+}}({\left[K^{+}\right]}_{i}-{\left[K^{+}\right]}_{s})\\ \;-\frac{dv}{vdt}{\left[K^{+}\right]}_{i}\\ \;\frac{d{\left[Cl^{-}\right]}_{i}}{dt}=-\frac{OV(-I_{vg6}+I_{vg5})}{F}-k_{Cl^{-}}({\left[Cl^{-}\right]}_{i}-{\left[Cl^{-}\right]}_{s})\\ \;-\frac{dv}{vdt}{\left[Cl^{-}\right]}_{i} \end{array}$$

Both voltage-gated channels and stretch-activated channels for calcium and potassium (Holdaway-Clarke and Hepler, 2003; Dutta and Robinson, 2004; Hepler et al., 2012) are included, while voltage-gated chloride channels are also included. We have also tested the inclusion of stretch-activated chloride channel, finding that inclusion of stretch-activated chloride channel can also reproduce all results in this work. The H^+^ ATPase pump and Cl^−^–2H^+^ symporter are also included (Gradmann and Hoffstadt, 1998; Gradmann, 2001; Shabala et al., 2006; Liu et al., 2010). Voltage-gated channels and pumps are described using a subscript vg, and the 6 voltage-gated transporters were studied in detail previously by us and others (Gradmann and Hoffstadt, 1998; Gradmann, 2001; Shabala et al., 2006; Liu et al., 2010). We note two important features of ion dynamics in pollen tube. First, parameter OV is surface (that is occupied by transporters) to volume ratio. When pollen tube grows, the total volume of pollen tube increases, but tip volume approximately remains about the same. This indicates that tip volume is continuously converted into shank volume (Liu et al., 2010). The mechanism of cell wall aging has recently been studied (Eggen et al., 2011; Kroeger and Geitmann, 2012). In Equation (10), OV is a constant for any volume size with a fixed radius of pollen tube, r, as pollen tube has a regular cylindrical shape (OV = 2/r where r is the radius of pollen tube). Therefore, ion dynamics is always the same for any chosen pollen tube tip volume with a fixed radius. Thus, our results shown in this work do not depend on the size of pollen tube tip. Second, current for transporters are proportional to membrane conductance (*I* = *g*(*V* − *E*) for ohmic relationship, and $I=gV\frac{[c_{i}]-[c_{e}]e^{-\frac{zV}{V_{ref}}}}{1-e^{-\frac{zV}{V_{ref}}}}$ for Goldman-Hodgkin-Katz constant-field relationship where g is membrane (Gradmann and Hoffstadt, 1998; Gradmann, 2001; Shabala et al., 2006; Liu et al., 2010). Therefore, parameter OV and membrane conductance always exist as a multiplying pair in Equation (10). This means that increasing OV and proportionally decreasing membrane conductance always lead to the same ion dynamics. Thus, our modeling results in this work are applicable to pollen tube with any pollen tube size and radius. This point reflects the following fact: when pollen tube radius increases, surface to volume ratio decreases. Therefore, for the same volume, the surface that is occupied by transporters decreases. As a result, increasing membrane conductance of all transporters by the same proportion to that of a decreased OV does not change ion dynamics.

Ion dynamics Equation (10) couples with hydrodynamics using Equation (4) and with cell wall dynamics using Equation (7).

#### Stretch-activated channels

While voltage-gated channels are activated by voltage, stretch-activated channels are activated by pressure (Dutta and Robinson, 2004; Hepler et al., 2012). Here we consider that stretch-activated *Ca*^2+^ and *K*^+^ channels are activated by cellular turgor. Equation (11) describes two states of stretch-activated channels.

(11) $$O\underset{k_{OC},k_{CO}}{⟷}C$$

where O and C are the completely open and completely closed state, *k*_*OC*_ and *k*_*CO*_ are the rate constants that control the transition between the open (O) and the closed (C) state, and they are functions of cellular turgor in the form of Equation (12).

(12) $$\begin{array}{l} k_{OC}=k_{OC}^{0}\\ k_{CO}=k_{CO}^{0}e^{k_{a}P} \end{array}$$

where *k*^0^_*OC*_ and *k*^0^_*CO*_ are the rate constants at zero turgor, and *k*_*a*_ > 0 is the strength for the channel activation by turgor. When turgor increases, it is more possible to open the channel.

When the stretch-activated *Ca*^2+^ and *K*^+^ channels are open, the transport of both *Ca*^2+^ and *K*^+^ are affected by voltage. Therefore, we use Goldman-Hodgkin-Katz constant-field relationship to describe the transport of both *Ca*^2+^ and *K*^+^ Equations (13) and (14).

For the stretch-activated *Ca*^2+^ channel, Equation (13) describes the transport of *Ca*^2+^.

(13) $$I_{sa1}=g_{Ca^{2+}}V\frac{{\left[Ca^{2+}\right]}_{i}-{\left[Ca^{2+}\right]}_{o}e^{-\frac{2V}{V_{ref}}}}{1-e^{-\frac{2V}{V_{ref}}}}$$

For the stretch-activated *K*^+^ channel, Equation (14) describes the transport of *K*^+^.

(14) $$I_{sa2}=g_{K^{+}}V\frac{{\left[K^{+}\right]}_{i}-{\left[K^{+}\right]}_{o}e^{-\frac{V}{V_{ref}}}}{1-e^{-\frac{V}{V_{ref}}}}$$

In Equations (13) and (14), *g*_*Ca*^2+^_ and *g*_*K*^+^_ are membrane conductance of *Ca*^2+^ and *K*^+^, respectively. $V_{ref}=\frac{RT}{F}$ with F being Faraday constant, R being gas constant and T is temperature.

#### Intrinsic coupling of hydrodynamics, cell wall and ion dynamics in pollen tube

Equations (1)–(14) couple hydrodynamics, cell wall and ion dynamics in pollen tube following kinetic and thermodynamic principles. When pollen tube growth is subject to any perturbation, pollen tube itself adjusts turgor, osmotic pressure, wall extensibility and concentration of four cellular ions, [*Ca*^2+^]_*i*_, [*H*^+^]_*i*_, [*K*^+^]_*i*_ and [*Cl*^−^]_*i*_ intrinsically.

#### Regulation coefficients of pollen tube growth

We adapt regulation analysis originally developed for metabolic networks (Rossell et al., 2006; Haanstra et al., 2008). Following Equation (1), when pollen tube growth is perturbed from one state (O1) to another state (O2), Equation (15) is always valid. An example of perturbations is that O1 state is for osmolarity in the media to be 0.36 Osm, and O2 state is for osmolarity in the media to be 0.18 Osm, Figure 6. Another example of perturbations is that O1 state is for equilibrium viscosity of wall materials to be 125 MPa s, and O2 state is for equilibrium viscosity of wall materials to be 225 MPa s, Figure 7.

(15) $$\begin{array}{l} {\bar{\left(\frac{d\nu }{dt}\right)}}_{O1}-{\bar{\left(\frac{d\nu }{dt}\right)}}_{O2}={\left(\bar{L_{p}A_{osm}\pi_{i}}\right)}_{O1}-{\left(\bar{L_{p}A_{osm}\pi_{i}}\right)}_{O2}\\ \;-\left({\left(\bar{L_{p}A_{osm}\pi_{o}}\right)}_{O1}-{\left(\bar{L_{p}A_{osm}\pi_{o}}\right)}_{O2}\right)\\ \;-\left({\bar{\left(L_{p}A_{osm}P\right)}}_{O1}-{\bar{\left(L_{p}A_{osm}P\right)}}_{O2}\right) \end{array}$$

The symbol X represents the average of X.

Following Equation (15), we have Equation (16).

(16) $$\begin{array}{l} \frac{{\left(\bar{L_{p}A_{osm}\pi_{i}}\right)}_{O1}-{\left(\bar{L_{p}A_{osm}\pi_{i}}\right)}_{O2}}{{\bar{\left(\frac{d\nu }{dt}\right)}}_{O1}-{\bar{\left(\frac{d\nu }{dt}\right)}}_{O2}}+\frac{-({\left(\bar{L_{p}A_{osm}\pi_{o}}\right)}_{O1}-{\left(\bar{L_{p}A_{osm}\pi_{o}}\right)}_{O2})}{{\bar{\left(\frac{d\nu }{dt}\right)}}_{O1}-{\bar{\left(\frac{d\nu }{dt}\right)}}_{O2}}\\ +\frac{-({\bar{\left(L_{p}A_{osm}P\right)}}_{O1}-{\bar{\left(L_{p}A_{osm}P\right)}}_{O2})}{{\bar{\left(\frac{d\nu }{dt}\right)}}_{O1}-{\bar{\left(\frac{d\nu }{dt}\right)}}_{O2}}=1 \end{array}$$

Therefore, we have

(17) $$R_{\pi_{i}}+R_{\pi_{o}}+R_{P}=1$$

with

(18) $$R_{\pi_{i}}^{w}=\frac{{\left(\bar{L_{p}A_{osm}\pi_{i}}\right)}_{O1}-{\left(\bar{L_{p}A_{osm}\pi_{i}}\right)}_{O2}}{{\bar{\left(\frac{d\nu }{dt}\right)}}_{O1}-{\bar{\left(\frac{d\nu }{dt}\right)}}_{O2}}$$

(19) $$R_{\pi_{0}}^{w}=-\frac{{\left(\bar{L_{p}A_{osm}\pi_{o}}\right)}_{O1}-{\left(\bar{L_{p}A_{osm}\pi_{o}}\right)}_{O2}}{{\bar{\left(\frac{d\nu }{dt}\right)}}_{O1}-{\bar{\left(\frac{d\nu }{dt}\right)}}_{O2}}$$

(20) $$R_{P}^{w}=-\frac{{\bar{\left(L_{p}A_{osm}P\right)}}_{O1}-{\bar{\left(L_{p}A_{osm}P\right)}}_{O2}}{{\bar{\left(\frac{d\nu }{dt}\right)}}_{O1}-{\bar{\left(\frac{d\nu }{dt}\right)}}_{O2}}$$

*R*^*w*^_π_*i*__, *R*^*w*^_π_*o*__ and *R*^*w*^_*P*_ are the regulation coefficients of cellular osmotic pressure, osmotic pressure in the media; and turgor pressure for water volume change in pollen tube, respectively.

Moreover, following Equation (2), we have

(21) $$\frac{dv}{vdt}=\frac{v_{ext}}{v}\phi (P-P_{c})\left(1+\frac{\frac{1}{\varepsilon }\frac{dP}{dt}}{\frac{v_{ext}}{v}\phi (P-P_{c})}\right)$$

Equation (21) leads to Equation (22).

(22) $$\text{​​}\log\frac{dv}{dt}\text{​}=\text{​}\log v_{ext}\phi +\log(P-P_{c})\text{​}+\text{​}\log\text{​}\left(\text{​}1+\frac{\frac{1}{\varepsilon }\frac{dP}{dt}}{\frac{v_{ext}}{v}\phi (P-P_{c})}\text{​}\right)$$

when pollen tube growth is perturbed from one state (O1) to another state (O2), Equation (23) is always valid.

(23) $$\begin{array}{l} {\left(\bar{\log\frac{dv}{dt}}\right)}_{O1}-{\left(\bar{\log\frac{dv}{dt}}\right)}_{O2}={\left(\bar{\log v_{ext}\phi }\right)}_{O1}-{\left(\bar{\log v_{ext}\phi }\right)}_{O2}\\ \;+{\bar{\left(\log(P-P_{c})\right)}}_{O1}-{\bar{\left(\log(P-P_{c})\right)}}_{O2}\\ \;+{\bar{\left(\log(1+\frac{\frac{1}{\varepsilon }\frac{dP}{dt}}{\frac{v_{ext}}{v}\phi (P-P_{c})})\right)}}_{O1}-{\bar{\left(\log(1+\frac{\frac{1}{\varepsilon }\frac{dP}{dt}}{\frac{v_{ext}}{v}\phi (P-P_{c})})\right)}}_{O2} \end{array}$$

Therefore, we have

(24) $$R_{\phi }^{c}+R_{P}^{c}+R_{o}^{c}=1$$

with

(25) $$R_{\phi }^{c}=\frac{{\left(\bar{\log\phi }\right)}_{O1}-{\left(\bar{\log\phi }\right)}_{O2}}{{\left(\bar{\log\frac{dv}{vdt}}\right)}_{O1}-{\left(\bar{\log\frac{dv}{vdt}}\right)}_{O2}}$$

(26) $$R_{P}^{c}=\frac{{\bar{\left(\log(P-P_{c})\right)}}_{O1}-{\bar{\left(\log(P-P_{c})\right)}}_{O2}}{{\left(\bar{\log(\frac{dv}{vdt}}\right)}_{O1}-\bar{\log{\left(\frac{dv}{vdt}\right)}_{O2}})}$$

(27) $$R_{O}^{c}\text{​​}=\text{​​}\frac{{\bar{\left(\log(1+\frac{\frac{1}{\varepsilon }\frac{dP}{dt}}{\frac{v_{ext}}{v}\phi (P-P_{c})})\right)}}_{O1}\text{​​​​}-{\bar{\left(\log(1+\frac{\frac{1}{\varepsilon }\frac{dP}{dt}}{\frac{v_{ext}}{v}\phi (P-P_{c})})\right)}}_{O2}}{{\left(\bar{\log\frac{dv}{dt}}\right)}_{O1}\text{​​​​}-{\left(\bar{\log\frac{dv}{dt}}\right)}_{O2}}$$

*R*^*c*^_ϕ_, *R*^*c*^_*P*_ and *R*^*c*^_*O*_ are the regulation coefficients of cellular wall extensibility, turgor pressure and temporal change in turgor pressure for cell wall chamber in pollen tube, respectively.

We note that, a regulation coefficient includes information at two levels. First, what is the specific relationship between a property such as turgor and volume change in pollen tube. Second, how a specific perturbation (e.g., osmolarity change in the media) has caused changes in both this property and volume change in pollen tube. For example, *R*^*w*^_*P*_ includes information at two levels. (1) If osmolarity in both the media and cell does not change, increasing turgor decreases water volume change Equation (1). (2) How a perturbation [e.g., osmolality change in the media (Figure 6) or change in the viscosity of wall material (Figure 7)] actually changes both turgor and water volume change. For example, in Figure 6, the increase in water volume change corresponds to the increase in turgor. This leads to a negative *R*^*w*^_*P*_, which implies that turgor negatively regulates water volume change. This is because Equation (1) indicates that increasing turgor should decrease water volume change. In Figure 7, the decrease in water volume change corresponds to the increase in turgor. As Equation (1) indicates that increasing turgor should decrease water volume change, this leads to a positive *R*^*w*^_*P*_, which implies that turgor positively regulates water volume change in this context.

In a similar manner, *R*^*c*^_ϕ_ also includes information at two levels. (1) If turgor does not change, increasing wall extensibility increases volume change in cell wall chamber Equation (2). (2) How a perturbation [e.g., osmolality change in the media (Figure 6) or change in the viscosity of wall material (Figure 7)] actually changes both wall extensibility and volume change in cell wall chamber. For example, in Figure 6, the increase of volume change in cell wall chamber corresponds to the increase in wall extensibility. This leads to a positive *R*^*c*^_ϕ_, which implies that cell wall extensibility positively regulates cell wall chamber volume change. This is because Equation (2) indicates that increasing cell extensibility should increase volume change in cell wall chamber. In Figure 7, the decrease of volume change in cell wall chamber corresponds to the decrease in wall extensibility. This also leads to a positive *R*^*c*^_ϕ_, which implies that cell wall extensibility positively regulates cell wall chamber volume. This is because Equation (2) indicates that decreasing cell extensibility should decrease volume change in cell wall chamber. However, Figure 7 also shows that the decrease of volume change in cell wall chamber corresponds to the increase in turgor. This leads to a negative *R*^*c*^_*P*_, which implies that turgor negatively regulates volume change in cell wall chamber. This is because Equation (2) indicates that increasing turgor should increase volume change in cell wall chamber.

### Parameters

The parameters and their link with all the above experiments are included in SI-Appendix. For those parameters available in the literature, we use their values. For example, the hydraulic conductivity of plant cell wall is ca. 1.0 × 10^−6^ m s^−1^ MPa^−1^ (Taiz and Zeiger, 2010). The vesicle fusion rate is described using *R* = *k*_2_ [*Ca*^2+^]_*i*_ where *k*_2_ = 4.1e–4 m s^−1^ M^−1^ based on experimental data Equation (7) (Roy et al., 1999; Blank et al., 2001; Kroeger et al., 2008). Then other parameter values are adjusted such that experimental oscillatory dynamics of pollen tube growth (Figure 2) (Messerli and Robinson, 2003; Zonia and Munnik, 2004, 2007, 2011; Zonia et al., 2006; Kroeger et al., 2011) is reproduced (Figure 2). Then, we use the parameters to make predictions and compare them with other experimental observations (Figures 2–5, 8). We note that, while the modeling equations must be formulated in specific forms following thermodynamic and kinetic principles, many parameter sets can be fitted against experimental data as the number of parameters is much more than that of experimental observations. By examining parameters randomly, we find that, when a parameter changes, if we allow at least one or more other parameters to change, we can find a new set of parameters that reproduces oscillatory dynamics (Figure 2) (Messerli and Robinson, 2003; Zonia and Munnik, 2004, 2007, 2011; Zonia et al., 2006; Kroeger et al., 2011). The model using the new set of parameters also makes correct predictions (Figures 2–5, 8). Therefore, the experimental observations can be reproduced using many parameter sets. In this sense, the model developed here is robust in that it reproduces experimental observations and makes correct predictions. In future, when a parameter is fitted using additional experimental data, one or more other parameters can also be simultaneously adjusted such that experimental observations (Benkert et al., 1997; Messerli and Robinson, 2003; Zonia and Munnik, 2004, 2007, 2011; Zonia et al., 2006; Szumlanski and Nielsen, 2009; Kroeger et al., 2011) can be reproduced.

### Conflict of interest statement

The authors declare that the research was conducted in the absence of any commercial or financial relationships that could be construed as a potential conflict of interest.
