# Supplementary material for: Dissecting the regulation of pollen tube growth by modeling the interplay of hydrodynamics, cell wall and ion dynamics
Source: Front Plant Sci. 2014 Aug 11;5:392. doi: 10.3389/fpls.2014.00392 (PMC4127481; doi:10.3389/fpls.2014.00392)
Supplement: Supplementary file 1 [file DataSheet1.DOCX]

**Supplementary information for “Dissecting the regulation of pollen tube growth by modelling the interplay of hydrodynamics, cell wall and ion dynamics”**

Junli Liu^*^ and Patrick J. Hussey^*^

School of Biological and Biomedical Sciences, Durham University, South Road, Durham DH1 3LE, UK

*Joint corresponding authors:

Patrick J. Hussey [p.j.hussey@durham.ac.uk](mailto:p.j.hussey@durham.ac.uk)

Junli Liu [junli.liu@durham.ac.uk](mailto:junli.liu@durham.ac.uk))

**Parameters and their links with experimental data**

Here we describe how experimental measurements are related to our model and parameters.

Table S1. Summary on parameters and how they are related to experimental measurements (when growth rate is calculated, pollen tube outer radius is set to be 5.0e-6 m).

| Equations | Parameters | References and their relevance to parameters |
| --- | --- | --- |
| Equation 1 | $L_{P}=1.0\times10^{-6}$m s^-1^ MPa^-1^ | The hydraulic conductivity of plant cell wall is ~$1.0\times10^{-6}$ m s^-1^ MPa^-1^ (Taiz and Zeiger, 2010).  Growth rate of in vitro-grown Arabidopsis pollen 4.07± 0.36 µm min^-1^ and 4.5 ±1.0 µm min^-1^, respectively, as model outputs for adjusting parameters (Ketelaar et al., 2008; Szumlanski and Nielsen , 2009).  With reference to an oscillatory period of ~50s at isotonic condition, hypertonic condition causes cell shrinking and longer oscillatory period (~100s) and hypotonic condition causes cell swelling and shorter oscillatory period (~25s) (Zonia and Munnik, 2004; 2011; Zonia et al. 2006). Qualitatively similar data are also in literature (Messerli and Robinson, 2003).  Knockout of Raba4d, a member of the Rab family of small GTPase proteins, decreases pollen tube length. Modelling predictions of pollen tube length are compared with these experiments (Szumlanski and Nielsen , 2009).  Changes in concentration of extracellular Ca^2+^ or *p*H changes oscillatory amplitude of pollen tube growth and the baseline growth rate. Modelling predictions of pollen tube growth rate are compared with these experiments (Messerli and Robinson, 2003).  Modelling predictions of water flow are compared with these experimental observations (Hill et al., 2012; Zonia et al., 2006). |
| Equation 2 | $P_{c}=0.2$ MPa  $\varepsilon$=10 MPa | Growth rate of in vitro-grown Arabidopsis pollen 4.07± 0.36 µm min^-1^ and 4.5 ±1.0 µm min^-1^, respectively, as model outputs for adjusting parameters (Ketelaar et al., 2008; Szumlanski and Nielsen, 2009).  Turgor: ~0.1- ~0.4 MPa. Change in turgor is small when extracellular osmolarity changes and increasing extracellular osmolarity slightly decreasing cellular turgor. Injection of oil changes turgor. No oscillatory changes in turgor were observed within a resolution limit of ca. 0.005 MPa. Modelling predictions of turgor and growth rate are compared with these experimental observations (Benkert et al., 1997).  ca. 0.1 MPa turgor change only changes growth rate by ca. 2 fold in sunflower leaves (Boyer, 2009).  Modelling predictions of turgor and growth rate are compared with these experimental observations. |
| Equation 3 | $[Osm]_{i}=0.5$M | Contribution of other molecules apart from ions to cellular osmolarity.  When other parameters are fixed in our model and for a fixed radius of 5.0e-6 m, increasing $[Osm]_{i}$increases growth rates. For example, for$[Osm]_{o}=0.36$M, growth rate of pollen tube is ca. 1.3µm/min; 2.4µm/min; 3.3µm/min; 4.4µm/min; 5.3 µm/min and 6.4 µm/min for $[Osm]_{i}=0.5$M; 0.6M; 0.7M, 0.8M; 0.9M and 1.0M respectively. For$[Osm]_{o}=0.18$M, growth rate of pollen tube is ca. 3.7µm/min; 4.7µm/min; 5.8µm/min, 6.9µm/min, 7.9 µm/min and 9.0 µm/min for $[Osm]_{i}=0.5$M; 0.6M; 0.7M, 0.8M; 0.9M and 1.0M respectively. Therefore, our model can simulate a wide range of growth rate for different combinations of extracellular and intracellular osmolarity conditions. |
| Equation 4 | $[Osm]_{o}$ | Contribution of other molecules apart from ions to extracellular osmolarity. Its change represents the change in media composition that affects osmolarity (Messerli and Robinson, 2003; Zonia and Munnik 2004; 2011; Zonia et al., 2006).  With reference to an oscillatory period of ~50s at isotonic condition, hypertonic condition causes longer oscillatory period (~100s) and hypotonic condition causes shorter oscillatory period (~25s) (Zonia and Munnik 2004; 2011; Zonia et al., 2006). Qualitatively similar data are also in literature (Messerli and Robinson, 2003).    Knockout of Raba4d, a member of the Rab family of small GTPase proteins, decreases pollen tube length. Modelling predictions of pollen tube length are compared with these experiments (Szumlanski and Nielsen, 2009).  Changes in concentration of extracellular Ca^2+^ or *p*H changes oscillatory amplitude of pollen tube growth and the baseline growth rate. Modelling predictions of pollen tube growth rate are qualitatively compared with these experiments (Messerli and Robinson, 2003).  Modelling predictions of water flow are compared with these experimental observations (Hill et al., 2012; Zonia et al. 2006). |
| Equation 5 | $P_{c}=0.2$ MPa  $\varepsilon$=10 MPa | Growth rate of in vitro-grown Arabidopsis pollen 4.07± 0.36 µm min^-1^ and 4.5 ±1.0 µm min^-1^, respectively, as model outputs for adjusting parameters (Ketelaar et al., 2008; Szumlanski and Nielsen, 2009).  Turgor: ~0.1- ~0.4 MPa. Change in turgor is small when extracellular osmolarity changes and increasing extracellular osmolarity slightly decreasing cellular turgor. Injection of oil changes turgor. No oscillatory changes in turgor were observed within a resolution limit of ca. 0.005 MPa. Modelling predictions of turgor and growth rate are compared with these experimental observations (Benkert et al., 1997).  ca. 0.1 MPa turgor change only changes growth rate by ca. 2 fold in sunflower leaves (Boyer, 2009).  Modelling predictions of turgor and growth rate are compared with these experimental observations. |
| Equation 6 and 7 | $\eta_{eq}=$ 25 MPa  $k_{1}$=0.01 s^-1^  $k_{2}=4.1\times10^{-4}$m s^-1^ M^-1^ | $k_{2}$is derived from the measured vesicle secretion rate (Blank et al., 2001; Kroeger et al., 2008; Roy et al., 1999). |
| Equation 8 and 9 | $r=5.0\times10^{-6}$m | Cell wall thickness is ~ 0.2µm-~0.5µm as model outputs for adjusting parameters (Holdaway-Clarke and Hepler 2003; Lancelle and Hepler 1988, 1992; McKenna et al. 2011).  The outer radius of a pollen tube is ~6 µm. (Kroeger et al., 2008, 2011). |
| Equation 10 | Each voltage-gated channel or pump has parameters relating to the specific transporter. See table S2 for details.  Stretch-activated channels for Ca^2+^ and K^+^. | Both voltage-gated channels and stretch-activated channels for Ca^2+^ and K^+^ are involved in pollen tube growth. Different types of transporters for Ca^2+^, K^+^, Cl^-^, H^+^ were experimentally identified.  These transporters are included in the model (Dutta and Robinson, 2004; Hepler, 2003; Gradmann, 2001; Gradmann and Hoffstadt, 1998 ; Holdaway-Clarke and Hepler et al., 2012; Liu et al., 2010; Shabala et al. 2006).  .  Oscillatory dynamics and $[Ca^{2+}]_{i}$=~5µM and $pH_{i}$=~6.8 as model outputs for adjusting parameters (Holdaway-Clarke and Hepler, 2003). |
| Equation 11-14 | stretch-activated $Ca^{2+}$channel: $g_{Ca^{2+}}=$  27 S m^-2^ M^-1^  $k_{CO}^{0}=$ 0.15 s^-1^, $k_{OC}^{0}=$ 0.15s^-1^  stretch-activated $K^{+}$channel: $g_{K^{+}}=$  540 S m^-2^ M^-1^  $k_{CO}^{0}=$ 0.15 s^-1^, $k_{OC}^{0}=$ 15s^-1^ | The outer radius of a pollen tube is ~6 µm (Kroeger et al. 2008, 2011).  Oscillatory dynamics in Ca^2+^, K^+^, Cl^-^, H^+^  and $[Ca^{2+}]_{i}$=~5µM and $pH_{i}$=~6.8 as model outputs for adjusting parameters (Dutta and Robinson, 2004; Hepler et al., 2012; Holdaway-Clarke and Hepler, 2003).  Growth rate of in vitro-grown Arabidopsis pollen 4.07± 0.36 µm min^-1^ and 4.5 ±1.0 µm min^-1^, respectively, as model outputs for adjusting parameters (Ketelaar et al., 2008; Szumlanski and Nielsen, 2009).  With reference to an oscillatory period of ~50s at isotonic condition, hypertonic condition causes cell shrinking and longer oscillatory period (~100s) and hypotonic condition causes cell swelling and shorter oscillatory period (~25s) (Zonia and Munnik, 2004; 2011; Zonia et al., 2006). Qualitatively similar data are also in literature (Messerli and Robinson, 2003). |

Table S2. Parameters for voltage-gated transporters at pollen tube tip. The principle and methodology for modelling voltage-gated transporters were previously described in detail (Gradmann 2001; Gradmann and Hoffstadt, 1998; Liu et al., 2010; Shabala et al. 2006).

| Transporter | Parameter |
| --- | --- |
| inward rectifying K^+^  channel | $g=800$S m^-2^ M^-1^, $k_{CO}^{0}=0.15$s^-1^,  $k_{OC}^{0}=15$s^-1^ |
| outward rectifying K^+^  channel | $g=430$ S m^-2^ M^-1^ , $k_{CO}^{0}=0.15$s^-1^ , $k_{OC}^{0}=0.15$ s^-1^ |
| Ca^2+^ channel | $g=800$ S m^-2^ M^-1^, $k_{CO}^{0}=0.15$s^-1^, $k_{OC}^{0}=0.15$  s^-1^ |
| H^+^ ATPase pump | $g=5.07$ S m^-2^, $k_{CO}^{0}=0.15s^{-1}$, $k_{OC}^{0}=1.5s^{-1}$ |
| Cl^-^-2H+ symporter | $g=4.05$S m^-2^, $k_{CO}^{0}=0.15$ s^-1^, $k_{OC}^{0}=0.15$ s^-1^ |
| Cl^-^ channel | $g=800$ S m^-2^ M^-1^, $k_{C_{1}O}^{0}=3.75$ s^-1^, $k_{OC_{1}}^{0}=0.075$ s^-1^,$k_{C_{2}O}^{0}=0.015$ s^-1^, $k_{OC_{2}}^{0}=0.15$ s^-1^ |

**References**

Benkert R, Obermeyer G and Bentrup FW (1997) The turgor pressure of growing lily pollen tubes. Protoplasma 198: 1–8.

Blank P, Vogel S, Malley J and Zimmerberg J (2001) A kinetic analysis of calcium-triggered exocytosis. J. Gen. Physiol. 118:145–156.

Boyer JS (2009) Cell wall biosynthesis and the molecular mechanism of plant enlargement. Functional Plant Biology 36: 383–394.

Dutta R and Robinson KR (2004) Identification and characterization of stretch-activated ion channels in pollen protoplasts. Plant Physiology 135: 1398-1406.

Gradmann D (2001) Models for oscillations in plants. Aust J Plant Physiol 28: 577–590.

Gradmann D and Hoffstadt J (1998) Electrocoupling of ion transporters in plants: interaction with internal ion ioncentrations. J. Membrane Biol 166: 51–59.

Hepler PK, Kunkel JG, Rounds CM and Winship LJ (2012) Calcium entry into pollen tubes. Trends Plant Sci. 17: 32-38.

Hill AE, Shachar-Hill B, Skepper JN, Powell J and Shachar-Hill Y (2012) An osmotic model of the growing pollen tube. PLoS One, 7: e36585.

Holdaway-Clarke TL and Hepler PK (2003) Control of pollen tube growth: role of ion gradients and fluxes. New Phytol 159: 539–563.

Ketelaar T, Galway ME, Mulder BM and Emons AM (2008) Rates of exocytosis and endocytosis in Arabidopsis root hairs and pollen tubes. J Microsc 231: 265–273.

Kroeger JH, Geitmann A and Grant M (2008) Model for calcium dependent oscillatory growth in pollen tubes. J Theor Biol 253: 363-374.

Kroeger J, Zerzour R and Geitmann A (2011) Regulator or driving force? The role of turgor pressure in oscillatory plant cell growth. PLoS One 6: e18549.

Lancelle, S.A., and Hepler, P.K. (1988). Cytochalasin-induced ultrastructural alterations in Nicotiana pollen tubes. Protoplasma (suppl. 2): 65–75.

Lancelle, S.A., and Hepler, P.K. (1992). Ultrastructure of freeze substituted pollen tubes of Lilium longiflorum. Protoplasma 167: 215–230.

Liu J, Piette BMAG, Deeks MJ, Franklin-Tong VE and Hussey PJ (2010) A compartmental model analysis of integrative and self-regulatory ion dynamics in pollen tube growth. PLoS One 5: e13157.

McKenna ST, Kunkel JG, Bosch M, Rounds CM, Vidali L, Winship LJ and Hepler PK (2009) Exocytosis precedes and predicts the increase in growth in oscillating pollen tubes. Plant Cell 21: 3026-3040.

Messerli MA and Robinson KR (2003) Ionic and osmotic disruption of the lily pollen tube oscillator: testing proposed models. Planta 217: 147-157.

Roy SJ, Holdaway-Clarke TL, Hackett GR, Kunkel JG, Lord EM, et al. (1999) Uncoupling secretion and tip growth in lily pollen tubes: evidence for the role of calcium in exocytosis. The Plant Journal 19: 379–386.

Shabala S, Shabala L, Gradmann D, Chen Z, Newman I, Mancus S (2006) Oscillations in plant membrane transport: model predictions, experimental validation, and physiological implications. J Exp Bot 57: 171-184.

Szumlanski AL and Nielsen E (2009) The Rab GTPase RabA4d regulates pollen tube tip growth in Arabidopsis thaliana. Plant Cell 21: 526–544.

Taiz L and Zeiger E (2010) Plant Physiology, Fifth Edition. Sinauer Associates. Sunderland, MA.

Zonia L and Munnik T (2004) Osmotically induced cell swelling versus cell shrinking elicits specific changes in phospholipid signals in tobacco pollen tubes. Plant Physiol. 134, 813–823.

Zonia L and Munnik T (2011) Understanding pollen tube growth: the hydrodynamic model versus the cell wall model. Trends Plant Sci. 16: 347–352.

Zonia L, Müller M and Munnik T (2006) Hydrodynamics and cell volume oscillations in the pollen tube apical region are integral components of the biomechanics of Nicotiana tabacum pollen tube growth. Cell Biochem. Biophys. 46: 209–232.
